# Diversity and characteristics of plant immunity–activating bacteria from *Brassicaceae* plants

**DOI:** 10.1186/s12866-023-02920-y

**Published:** 2023-07-05

**Authors:** Hiroki Kaneko, Fuma Miyata, Mari Kurokawa, Kenji Hashimoto, Kazuyuki Kuchitsu, Toshiki Furuya

**Affiliations:** Department of Applied Biological Science, Faculty of Science and Technology, Tokyo University of Science, Yamazaki, Noda, 2641, 278-8510 Chiba Japan

**Keywords:** Biocontrol, Brassicaceae, Cultured plant cells, Endophyte, Induced systemic resistance, Plant immunity, Priming, Reactive oxygen species

## Abstract

**Background:**

Microorganisms that activate plant immune responses are useful for application as biocontrol agents in agriculture to minimize crop losses. The present study was conducted to identify and characterize plant immunity–activating microorganisms in *Brassicaceae* plants.

**Results:**

A total of 25 bacterial strains were isolated from the interior of a *Brassicaceae* plant, *Raphanus sativus* var. *hortensis*. Ten different genera of bacteria were identified: *Pseudomonas*, *Leclercia*, *Enterobacter*, *Xanthomonas*, *Rhizobium*, *Agrobacterium*, *Pantoea*, *Rhodococcus*, *Microbacterium*, and *Plantibacter*. The isolated strains were analyzed using a method to detect plant immunity–activating microorganisms that involves incubation of the microorganism with tobacco BY-2 cells, followed by treatment with cryptogein, a proteinaceous elicitor of tobacco immune responses. In this method, cryptogein-induced production of reactive oxygen species (ROS) in BY-2 cells serves as a marker of immune activation. Among the 25 strains examined, 6 strains markedly enhanced cryptogein-induced ROS production in BY-2 cells. These 6 strains colonized the interior of *Arabidopsis* plants, and *Pseudomonas* sp. RS3R-1 and *Rhodococcus* sp. RS1R-6 selectively enhanced plant resistance to the bacterial pathogens *Pseudomonas syringae* pv. *tomato* DC3000 and *Pectobacterium carotovorum* subsp. *carotovorum* NBRC 14082, respectively. In addition, *Pseudomonas* sp. RS1P-1 effectively enhanced resistance to both pathogens. We also comprehensively investigated the localization (i.e., cellular or extracellular) of the plant immunity–activating components produced by the bacteria derived from *R. sativus* var. *hortensis* and the components produced by previously isolated bacteria derived from another *Brassicaceae* plant species, *Brassica rapa* var. *perviridis*. Most gram-negative strains enhanced cryptogein-induced ROS production in BY-2 cells via the presence of cells themselves rather than via extracellular components, whereas many gram-positive strains enhanced ROS production via extracellular components. Comparative genomic analyses supported the hypothesis that the structure of lipopolysaccharides in the outer cell envelope plays an important role in the ROS-enhancing activity of gram-negative *Pseudomonas* strains.

**Conclusions:**

The assay method described here based on elicitor-induced ROS production in cultured plant cells enabled the discovery of novel plant immunity–activating bacteria from *R. sativus* var. *hortensis*. The results in this study also suggest that components involved in the ROS-enhancing activity of the bacteria may differ depending largely on genus and species.

**Supplementary Information:**

The online version contains supplementary material available at 10.1186/s12866-023-02920-y.

## Introduction

Biological control of plant diseases using beneficial microorganisms has received considerable attention as a promising alternative to the use of pesticides, which exert potential adverse effects on both human health and soil microbial communities [1, 2]. A variety of pathogens attack plants in the environment, and in agriculture, this can lead to significant crop losses. Beneficial microorganisms protect plants from pathogens via several different mechanisms, including the production of antimicrobial compounds, competition with pathogens for space and nutrients, and activation of plant immune responses [3, 4]. Microorganisms that activate plant immune responses are useful for application as biocontrol agents in agriculture, as they function like vaccines in plants without causing unwanted adverse effects [5, 6]. Pathogen recognition by plants leads to the initiation of defense responses, including the generation of reactive oxygen species (ROS), the expression of various defense-related genes, and the biosynthesis of phytoalexins and defense hormones [7–9]. Several types of plant-associated microorganisms can activate the plant immune system through a phenomenon known as induced systemic resistance (ISR) [10], which enables plants to engage more-rapid and stronger defense responses with no or low growth inhibition.

ISR mediated by plant-associated bacteria belonging to the genera *Pseudomonas* and *Bacillus* has been well studied to date [11–14]. For example, ISR in several plant species such as *Arabidopsis* and carnation can be provoked by the rhizobacterium *Pseudomonas fluorescens* WCS417r [15, 16]. The rhizobacterium *Bacillus cereus* AR156 confers resistance in *Arabidopsis* to the bacterial pathogen *Pseudomonas syringae* pv. *tomato* and the fungal pathogen *Botrytis cinerea* [17, 18]. In addition, endophytes are suitable for use as biocontrol agents because of their inherent ability to stably colonize the interior of plants [3, 4, 19, 20]. For example, the endophytic bacterium *Streptomyces* sp. EN27 can induce resistance in *Arabidopsis* to the bacterial pathogen *Pectobacterium carotovorum* subsp. *carotovorum* and the fungal pathogen *Fusarium oxysporum* via ISR [21, 22]. Pretreatment of *Arabidopsis* with the well-characterized endophytic bacterium *Paraburkholderia phytofirmans* PsJN increases resistance to *P. syringae* pv. *tomato* [23, 24]. Recent reports indicate that *Azospirillum* sp. B510, an endophytic bacterium isolated from rice, induces disease resistance in rice and tomato [25, 26]. Identifying the different types of plant immunity–activating bacteria that inhabit plants would not only enhance understanding of plant-microbe interactions in nature but could also facilitate the application of these microorganisms as biocontrol agents.

Conventional methods to screen for plant immunity–activating bacteria are based on monitoring disease symptoms using whole plants and pathogens. However, these methods are cumbersome and tend to be laborious and time consuming. We have established a method using cultured plant cells to directly detect microorganisms that activate the plant immune system based on plant-microbe interactions [27]. In this method, tobacco BY-2 cells are incubated with a microorganism and then treated with cryptogein, a proteinaceous elicitor of tobacco immune responses secreted by the pathogenic oomycete *Phytophthora cryptogea* [28–34]. Cryptogein-induced production of ROS in BY-2 cells serves as a marker to assess the potential of a microorganism to activate the plant’s defense response. This method increases throughput in screening for microorganisms that “prime” and potentiate plant immune responses, and its use led to the discovery of novel plant immunity–activating bacterial endophytes from a *Brassicaceae* plant, *Brassica rapa* var. *perviridis* [27].

In the present study, we isolated endophytes from another *Brassicaceae* plant species, *Raphanus sativus* var. *hortensis*. We were interested in whether plant immunity–activating bacteria could be obtained from other plants of the same family, and whether there were differences in the types of plant immunity–activating bacteria. A total of 25 bacterial strains isolated from the plant interior were assayed using the described detection method, and strains that enhanced cryptogein-induced ROS production in BY-2 cells were selected. After selection of the plant immunity–activating bacteria, 3 endophytes that induce bacterial pathogen resistance in whole *Arabidopsis* plants were identified. We also report here the characterization of the components involved in plant immune activation produced by bacteria obtained from the 2 *Brassicaceae* plant species.

## Results

### Isolation of bacteria from the interior of ***R. sativus var. hortensis*** plants

**Fig. 1:**
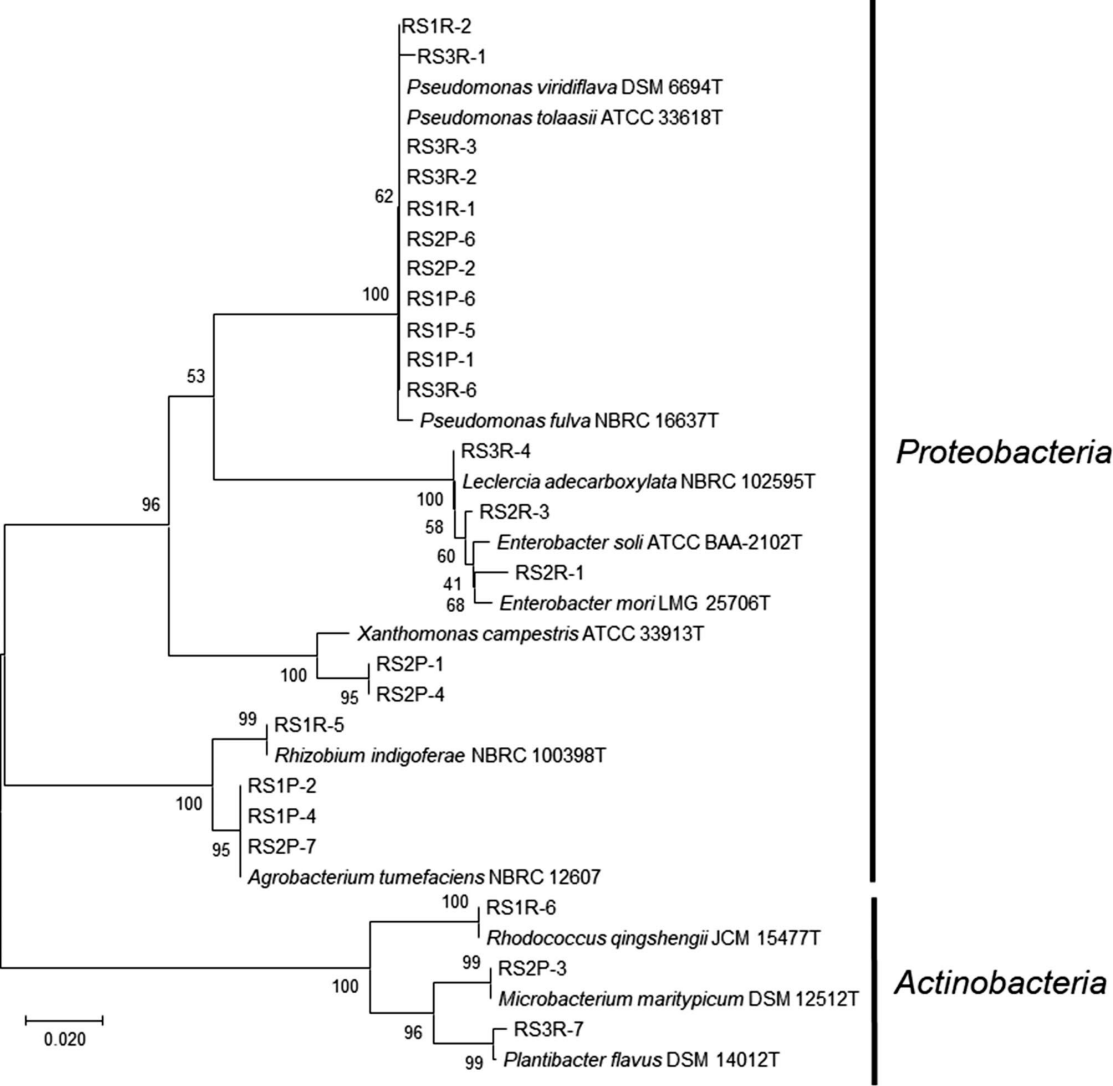
Phylogenetic relationships of bacterial strains recovered from the interior of *R. sativus* var. *hortensis* plants based on 16 S rRNA gene sequences. Bootstrap values from 1000 replications are shown at each of the branch points on the tree. Strains RS1R-3 and RS1R-4 were not included in the phylogenetic tree because the region of the 16 S rRNA gene sequence read in these strains differed from that of the other strains, as described in the Materials and Methods

Microorganisms were isolated from the interior of *R. sativus var. hortensis* plants. Petioles and roots of the plants (Fig. S1) were surface-sterilized and placed on NBRC802 and ISP2 agar plates, as described in the Materials and Methods [27]. A total of 25 bacterial strains were isolated, of which 11 and 14 strains were derived from petioles and roots, respectively (Table S1). Taxonomic identification based on 16 S rRNA gene sequencing revealed that these bacteria belonged to 10 different genera: *Pseudomonas*, *Leclercia*, *Enterobacter*, *Xanthomonas*, *Rhizobium*, *Agrobacterium*, *Pantoea*, *Rhodococcus*, *Microbacterium*, and *Plantibacter* (Table S1 and Fig. 1). These strains were further divided into 2 phyla, *Proteobacteria* and *Actinobacteria* (Fig. 1). Interestingly, 21 strains were classified as *Proteobacteria*, and 11 of these *Proteobacteria* strains belonged to the genus *Pseudomonas* (Fig. 1).

### Assay of bacterial ability to prime plant immune responses

The relationship between the immune responses of tobacco BY-2 cells and the pathogenic oomycete–derived elicitor cryptogein has been well characterized [28–34]. Cryptogein triggers various immune responses in BY-2 cells, including ROS production. Using BY-2 cells and cryptogein, we previously established a method to directly detect microorganisms that activate the plant immune system (Fig. S2) [27]. This method involves incubation of a microorganism with BY-2 cells, followed by treatment with cryptogein and quantitative analysis of ROS production via chemiluminescence. In this process, before the addition of cryptogein, microorganism-treated BY-2 cells are collected and suspended in fresh buffer to remove metabolites derived from both microbial cells and BY-2 cells (e.g., organic compounds, ROS, and ROS scavengers). If a microorganism is capable of priming the immune response of BY-2 cells, pretreatment of the cells with that microorganism will enhance cryptogein-induced ROS production.

**Fig. 2:**
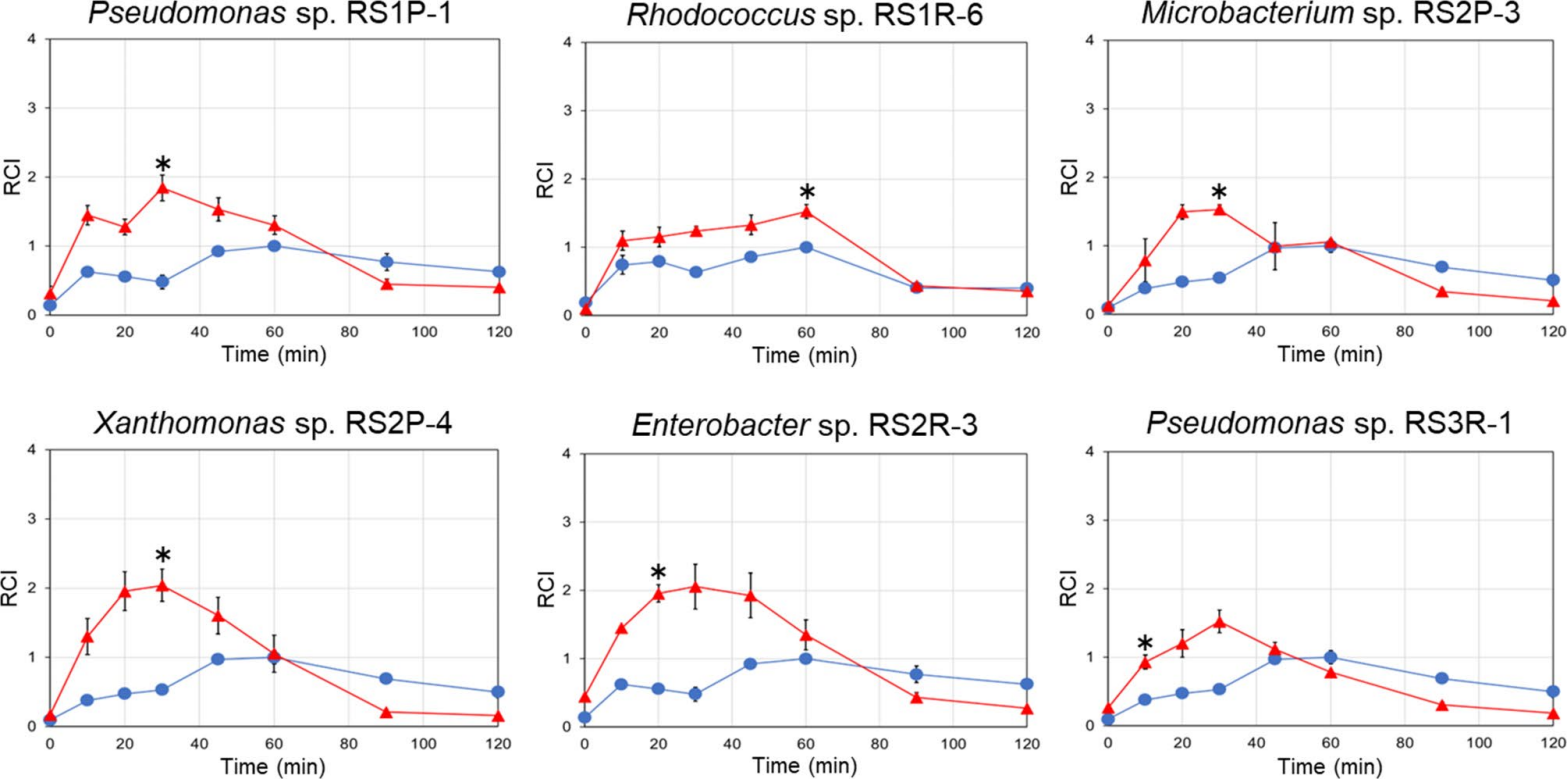
Cryptogein-induced ROS production in BY-2 cells co-incubated with bacteria. Bacteria that enhanced cryptogein-induced ROS production are shown. BY-2 cells were co-incubated with bacteria of each strain (∆) or subjected to mock treatment (only a mixture of medium and buffer, ○), and then cryptogein was added. ROS production was monitored based on chemiluminescence. The maximum value of the mock control was expressed as 1.0, and relative chemiluminescence intensity (RCI) is shown. Average values ± SE from three independent experiments are presented. Asterisks indicate a significant difference from the mock control based on Student’s t-test (*, P < 0.05)

In this study, bacterial endophytes isolated from *R. sativus var. hortensis* plants were subjected to the assay to identify those capable of priming the plant immune response. Most of the isolated bacteria (19 strains) exhibited no or only minor effects on BY-2 cells during co-incubation (Fig. S3), but 6 strains markedly enhanced cryptogein-induced ROS production by the BY-2 cells (Fig. 2): *Pseudomonas* sp. RS1P-1, *Rhodococcus* sp. RS1R-6, *Microbacterium* sp. RS2P-3, *Xanthomonas* sp. RS2P-4, *Enterobacter* sp. RS2R-3, and *Pseudomonas* sp. RS3R-1. It is interesting to note that these plant immunity–activating bacteria belonged to distinct phylogenetic clusters (Fig. 1).

### Biocontrol activity of selected bacteria

**Fig. 3:**
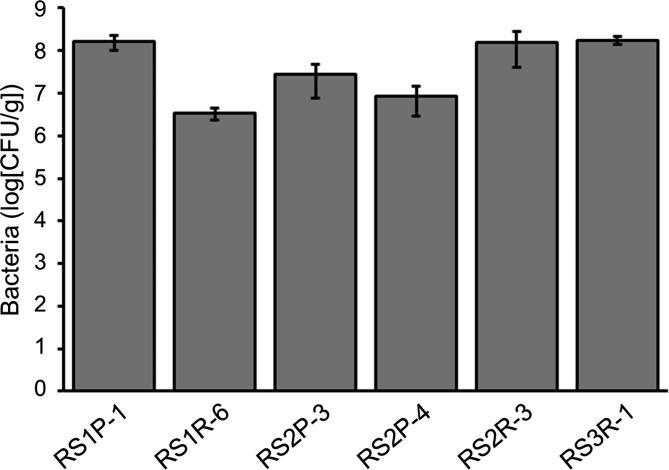
Colonization of *Arabidopsis* plants by selected bacteria. Plants were inoculated with each strain of selected bacteria by immersing the root tip of 7-day-old seedlings in bacterial cell culture solution, followed by cultivation for 7 days. After extracts of surface-sterilized plants were plated on medium, the number of colonies that formed on the plate was determined. No colonies were formed for plants that received mock treatment (only medium) instead of bacterial cell culture solution. Average values ± SE from three independent experiments are presented

The selected bacteria were subjected to the assay using whole *Arabidopsis* plants. Each selected strain was inoculated into plants by immersing the root tip of seedlings into bacterial cell culture solution. We observed that 5 strains (RS1P-1, RS1R-6, RS2P-3, RS2P-4, and RS3R-1) had no effect on plant growth after inoculation, whereas the remaining strain (RS2R-3) significantly reduced plant growth after inoculation (Fig. S4). These strains colonized the interior of the *Arabidopsis* plants (Fig. 3). The number of bacteria ranged from 10^6^ to 10^8^ colony forming unit (CFU) per gram of *Arabidopsis* plant tissue, depending on the bacterial strain.

**Fig. 4:**
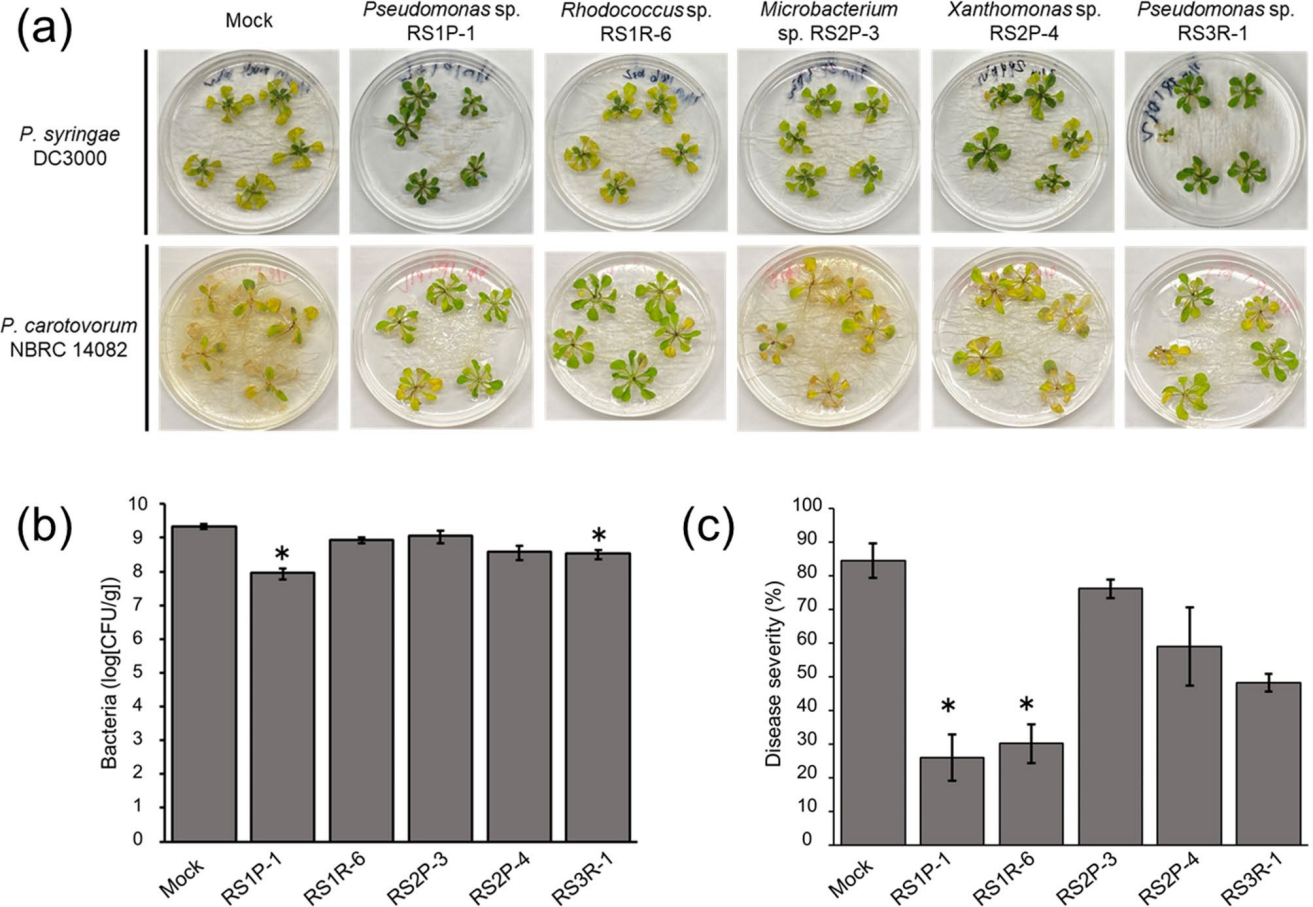
Pathogen resistance of *Arabidopsis* plants pretreated with selected bacteria. RS1P-1–, RS1R-6–, RS2P-3–, RS2P-4–, RS3R-1–, or mock (only medium)–treated *Arabidopsis* seedlings were cultivated for 7 days, and the plants were then challenged with *P. syringae* pv. *tomato* DC3000 or *P. carotovorum* subsp. *carotovorum* NBRC 14082 and cultivated for an additional 4 days. (a), representative photographs. (b), proliferation of strain DC3000. After plating extracts of surface-sterilized aerial tissues of plants on medium, the number of colonies of strain DC3000 that formed on the plate was determined. (c), severity of disease caused by strain NBRC 14082. Disease severity is indicated as a percentage calculated by dividing the number of damaged leaves by the number of all leaves. Average values ± SE from three independent experiments are presented. Asterisks indicate a significant difference from the mock control based on Student’s *t*-test (*, P < 0.05)

*Arabidopsis* seedling treated with each strain of the 5 endophytes was challenged with the hemibiotrophic bacterial pathogen *Pseudomonas syringae* pv. *tomato* DC3000. Mock-treated plants exhibited symptoms of severe chlorosis (Fig. 4a). In contrast, pretreatment with strains RS1P-1 and RS3R-1 resulted in significantly milder disease symptoms in plants compared to mock-treated plants (Fig. 4a). The density of strain DC3000 in *Arabidopsis* plants decreased to 4% and 15% following treatment with strains RS1P-1 and RS3R-1, respectively, compared with mock-treated plants (Fig. 4b). Similarly, although plants challenged with the necrotrophic bacterial pathogen *Pectobacterium carotovorum* subsp. *carotovorum* NBRC 14082 exhibited soft rot, pretreatment with strains RS1P-1 and RS1R-6 significantly reduced plant disease symptoms (Fig. 4a and c). Notably, *Pseudomonas* sp. RS3R-1 and *Rhodococcus* sp. RS1R-6 selectively enhanced plant resistance to *P. syringae* pv. *tomato* DC3000 and *P. carotovorum* subsp. *carotovorum* NBRC 14082, respectively. Furthermore, *Pseudomonas* sp. RS1P-1 effectively enhanced resistance to both pathogens.

### Characterization of components that promote ROS production

We comprehensively characterized the components involved in plant immune activation produced by 14 strains derived from the 2 types of *Brassicaceae* plants. (Table 1). We used the ROS-enhancing strains isolated from *B. rapa* var. *perviridis* in our previous study [27] in addition to the ROS-enhancing strains isolated from *R. sativus* var. *hortensis* in this study to obtain more information on the plant immunity–activating components. We first evaluated the thermal stability of the components. Bacterial cell culture medium was autoclaved, and cryptogein-induced ROS were measured. As expected, the ROS-enhancing activity of most of the strains (11 strains) was lost after autoclaving (Table 1 and Fig. S5). However, surprisingly, the activity of *Delftia* sp. BR1R-2, *Bacillus* sp. BR2S-4, and *Rhodococcus* sp. RS1R-6 was retained after autoclaving (Table 1 and Fig. S5). These results indicate that the components responsible for the ROS-enhancing activity of these 3 strains are highly thermostable.

**Table 1:** Characteristics of components enhancing cryptogein-induced ROS production

Gram staining	Genus	Strain ^a^	Localization ^b^		Thermal stability ^c^
			Cellular	Extracellular	
–	*Enterobacter*	RS2R-3	+	**–**	**–**
–	*Pseudomonas*	BR1R-3	+	**–**	**–**
–	*Pseudomonas*	BR1R-5	+	**–**	**–**
–	*Pseudomonas*	RS1P-1 ^d^	+	**–**	**–**
–	*Pseudomonas*	RS3R-1 ^d^	+	**–**	**–**
–	*Xanthomonas*	RS2P-4	+	**–**	**–**
–	*Delftia*	BR1R-2 ^d^	+	**–**	+
–	*Agrobacterium*	BR3S-1	+	**+**	**–**
+	*Paenarthrobacter*	BR3S-9	*+*	**–**	**–**
+	*Bacillus*	BR2S-4	+	**–**	+
+	*Arthrobacter*	BR2S-6 ^d^	+	+	**–**
+	*Bacillus*	BR2R-4	+	+	**–**
+	*Microbacterium*	RS2P-3	+	+	**–**
+	*Rhodococcus*	RS1R-6 ^d^	+	+	+

^a^ BR strains were isolated from *B. rapa* var. *perviridis* in our previous study [27] and RS strains were isolated from *R. sativus* var. *hortensis* in this study

^b^ Bacterial cell culture solution was centrifuged to separate the cells and extracellular components before measurement of cryptogein-induced ROS production

^c^ Bacterial cell culture solution was autoclaved before the measurement

^d^ These strains colonized the interior of the *Arabidopsis* plants without affecting plant growth and induced whole-plant resistance to *P. syringae* pv. *tomato* DC3000 and/or *P. carotovorum* subsp. *carotovorum* NBRC 14082

We also investigated the localization of the components. Bacterial cell culture medium was centrifuged to separate the cells and extracellular components, and cryptogein-induced ROS production was assayed. Interestingly, the cellular fraction of 7 of the 8 gram-negative strains (1 *Enterobacter* strain, 4 *Pseudomonas* strains, 1 *Xanthomonas* strain, and 1 *Delftia* strain) exhibited ROS-enhancing activity, but the extracellular component fraction did not (Table 1 and Fig. S6). These results suggest that components associated with the cell envelope are involved in the ROS-enhancing activity of these 7 gram-negative bacteria. In contrast, extracellular components exhibited ROS-enhancing activity for 4 of the 6 gram-positive strains (*Arthrobacter* sp. BR2S-6, *Bacillus* sp. BR2R-4, *Microbacterium* sp. RS2P-3, and *Rhodococcus* sp. RS1R-6) (Table 1 and Fig. S6). These results indicate that the bacterial components responsible for ROS-enhancing activity vary greatly between genera and species.

### Comparative genomic analysis of ***Pseudomonas*** strains

We observed that some strains of gram-negative *Pseudomonas* enhanced cryptogein-induced ROS production in BY-2 cells (Table 1), whereas other strains did not (Fig. S3). Assuming that the difference was at the genome level, we analyzed genetic features associated with the ROS-enhancing activity of the *Pseudomonas* strains by comparative genomic analysis. The genome sequences of 4 strains (BR1R-3, BR1R-5, RS1P-1, and RS3R-1) that enhanced ROS production (as shown in Table 1) have already been determined [35]. The genome sequence of strain RS3R-2, which did not enhance ROS production (Fig. S3), has also been determined [35]. Among the 10 *Pseudomonas* strains in the NBRC (NITE Biological Resource Center, Japan) culture collection for which the genomes have been sequenced, 3 strains (NBRC 13583, NBRC 14167, and NBRC 102411) did not enhance cryptogein-induced ROS production in BY-2 cells (Fig. S7). We therefore performed a comparative genomic analysis of 4 strains that exhibited ROS-enhancing activity (BR1R-3, BR1R-5, RS1P-1, and RS3R-1) and 4 strains that did not exhibit such activity (RS3R-2, NBRC 13583, NBRC 14167, and NBRC 102411).

**Fig. 5:**
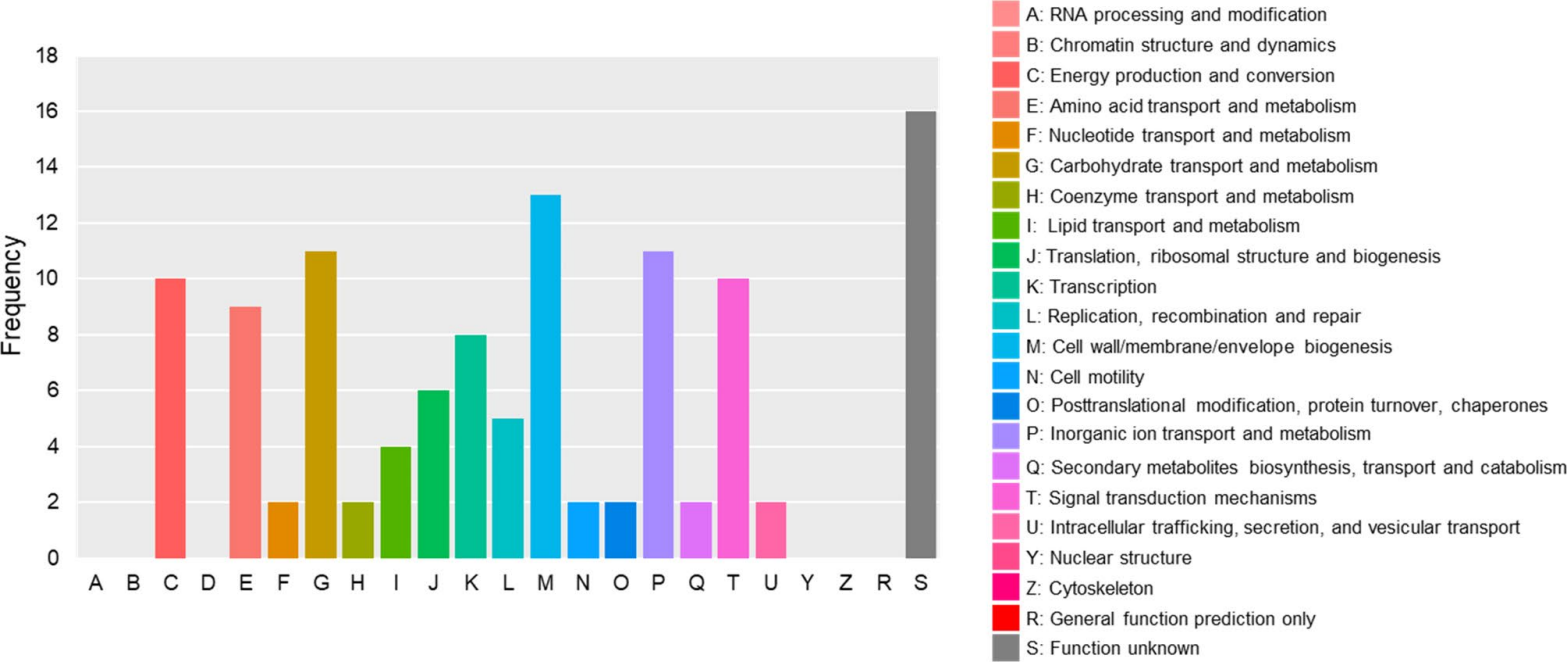
Functional classification of clusters of orthologous genes present in all ROS-enhancing strains that were absent in all non–ROS-enhancing strains. Clusters of orthologous genes from the *Pseudomonas* genomes were listed, and the list was filtered by the clusters present in all strains that enhanced cryptogein-induced ROS production in BY-2 cells (BR1R-3, BR1R-5, RS1P-1, and RS3R-1) but absent in all non–ROS-enhancing strains (RS3R-2, NBRC 13583, NBRC 14167, and NBRC 102411). The capital letters in x-axis indicates the COG categories as listed on the right of the histogram and the y-axis indicates the number of genes

**Fig. 6:**
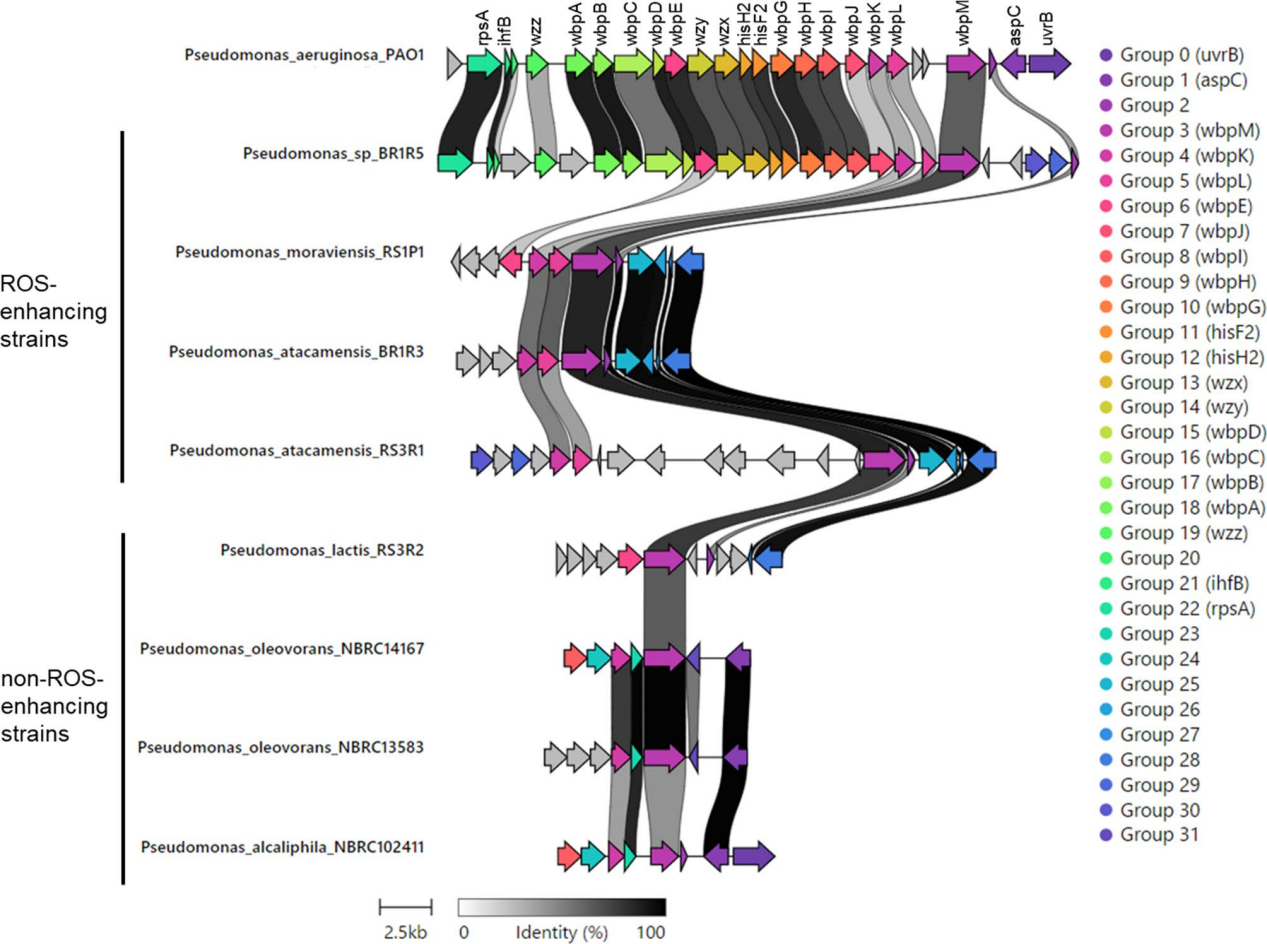
Comparative analysis of gene clusters responsible for synthesis of the *O*-specific antigen of LPS in *Pseudomonas* strains using cblaster v1.3.8. Links between homologous genes are shown using specific colors. The gene cluster of *P. aeruginosa* PAO1, which has been well characterized [36], is shown for comparison

We identified 102 clusters of orthologous genes present in all ROS-enhancing strains that were absent in all non–ROS-enhancing strains (Table S2 and Fig. S8). These clusters were classified based on function (Fig. 5). Notably, cell wall/membrane/envelope biogenesis (M) was the most common category, which was consistent with the results of analyses indicating that ROS-enhancing components were associated with the cell envelope of gram-negative *Pseudomonas* strains (Table 1). In particular, COG0472 of category M corresponds to the gene *wbpL* (Table S2), which encodes a glycosyltransferase required for the synthesis of the *O*-specific antigen of lipopolysaccharides (LPSs) in the outer cell envelope of *Pseudomonas* strains [36]. In addition, the gene clusters responsible for synthesis of the *O*-specific antigens of LPSs of the 8 *Pseudomonas* strains were analyzed using cblaster v1.3.8, a tool for identifying clusters of co-localized homologous sequences. We found differences in the structures of the gene clusters for LPS biosynthesis (the *O*-specific antigen gene clusters) including *wbpL* between strains that did and did not exhibit ROS-enhancing activity (Fig. 6). These results suggest that differences in the LPS structure play important roles in determining the ROS-enhancing activity of *Pseudomonas* strains.

## Discussion

Plant immunity–activating microorganisms have attracted considerable attention due to their ability to induce pathogen resistance. We previously established a method to directly detect microorganisms that activate the plant immune system by monitoring cryptogein-induced ROS production in BY-2 cells as a marker of immune activation [27]. By applying this method to 31 bacterial endophytes isolated from *B. rapa* var. *perviridis*, 8 strains that enhance cryptogein-induced ROS production were obtained. Of these strains, *Delftia* sp. BR1R-2 and *Arthrobacter* sp. BR2S-6 induced whole-plant resistance to the bacterial pathogens *P. syringae* pv. *tomato* DC3000 and *P. carotovorum* subsp. *carotovorum* NBRC 14082. We also found that pathogen-induced expression of plant defense-related genes was enhanced by pretreatment with strain BR1R-2 [27].

In this study, we first isolated endophytes from another *Brassicaceae* plant species, *R. sativus* var. *hortensis*. A total of 25 bacterial strains were isolated, of which 21 and 4 of the strains were classified as *Proteobacteria* and *Actinobacteria*, respectively (Fig. 1 and Table S1). Bacterial endophytes are generally classified within 4 phyla: *Proteobacteria*, *Actinobacteria*, *Firmicutes*, and *Bacteroidetes* [37, 38]. In our previous study, we found that 31 bacterial endophytes isolated from *B. rapa* var. *perviridis* belonged to 3 phyla, *Proteobacteria* (12 strains), *Actinobacteria* (8 strains), and *Firmicutes* (11 strains) [27]. In the present study, by contrast, no *Firmicutes* strains were isolated, and *Proteobacteria* strains dominated the isolated endophytes. The *B. rapa* var. *perviridis* and *R. sativus* var. *hortensis* plants used in these studies were grown using a similar method on the same farm, suggesting that the observed differences in the microbiome are partly due to differences in the host plants. However, other factors such as soil sampling time and soil conditions can also influence the microbiome. Sun and coworkers recently used a 16 S rRNA metagenomic approach to thoroughly probe the *R. sativus* microbiome [39]. They have reported that the dominant endophytic bacteria in *R. sativus* were *Proteobacteria*, *Bacteroidetes*, and *Actinomycetes* at the phylum level irrespective of cultivation conditions including greenhouse and open field cultivation. In *Proteobacteria* phylum, *Pseudomonas*, *Brevundimonas*, and *Cellvibrio* had higher abundances in *R. sativus* at the genus level [39]. In our present study, 21 strains were classified as *Proteobacteria*, and 11 of these *Proteobacteria* strains belonged to the genus *Pseudomonas* (Fig. 1). *Pseudomonas* strains have been isolated from *R. sativus* also in other studies [40, 41], suggesting that strains of this genus might play an important role in *R. sativus*.

The bacteria isolated from *R. sativus* var. *hortensis* were assayed for the ability to prime plant immune responses. Among the 25 strains of isolated bacteria, 6 strains markedly enhanced cryptogein-induced ROS production in BY-2 cells (Fig. 2 and S3). Furthermore, each selected bacterial strain was inoculated into whole *Arabidopsis* plants before pathogen infection (Figs. 3 and 4). Strains RS3R-1 and RS1R-6 enhanced the resistance of *Arabidopsis* plants to challenges with *P. syringae* pv. *tomato* DC3000 and *P. carotovorum* subsp. *carotovorum* NBRC 14082, respectively. Furthermore, strain RS1P-1 enhanced the resistance of *Arabidopsis* plants to both pathogens. These results demonstrate that the assay method based on elicitor-induced ROS production in cultured plant cells is useful for identifying various types of microorganisms that activate plant defense responses. Although the other strains examined did not enhance the resistance of *Arabidopsis* to *P. syringae* pv. *tomato* DC3000 and *P. carotovorum* subsp. *carotovorum* NBRC 14082, it is possible that these strains might enhance the resistance of other plant species to other pathogens.

We identified 2 *Pseudomonas* strains (RS1P-1 and RS3R-1) and 1 *Rhodococcus* strain (RS1R-6) that enhanced the pathogen resistance of plants (Fig. 4). Plant-associated bacteria of the genus *Pseudomonas* have been well-characterized to date [12, 14]. In addition to *P. fluorescens* WCS417r, which was described in the Introduction Section [15, 16], *P. fluorescens* CHA0, *P. putida* WCS358, and *P. aeruginosa* 7NSK2 reportedly trigger ISR in plants [42–44]. Additionally, a few strains of the genus *Rhodococcus* reportedly exhibit biocontrol activity. *Rhodococcus erythropolis* R138 prevents the bacterial pathogen *Pectobacterium atrosepticum* from infecting potato tubers by degrading a compound required for quorum sensing by this pathogen [45]. *Rhodococcus* sp. KB6, an endophytic bacterium isolated from *Arabidopsis*, enhances sweet potato resistance to black rot disease caused by *Ceratocystis fimbriata* [46]. Using cultured plant cells, in the present study, we confirmed that strains RS1P-1, RS3R-1, and RS1R-6 activate the plant immune system, and detailed characterizations of the biocontrol mechanisms of these strains are currently underway.

We also comprehensively investigated whether the plant immunity–activating components associated with the 14 bacterial strains derived from the 2 types of *Brassicaceae* plants were cellular or extracellular (Table 1). Notably, the cells of 7 of the 8 gram-negative strains enhanced cryptogein-induced ROS production in BY-2 cells, but extracellular components produced by these strains did not (Table 1 and Fig. S6). Because intracellular bacterial components cannot make direct contact with plant cells, we hypothesized that the components responsible for the ROS-enhancing activity in these gram-negative bacteria are associated with the cell envelope. LPS is an abundant component of the outer cell envelope of gram-negative bacteria and is known to play important roles in triggering immune responses in plants [47]. LPS of *Pseudomonas* strains reportedly induces resistance to *Fusarium* wilt in carnation and radish [16, 48]. Furthermore, the results of a comparative genomic analysis supported the hypothesis that LPS plays an important role in enhancing ROS production by the gram-negative *Pseudomonas* strains examined in this study (Figs. 5 and 6). We found that all of the ROS-enhancing strains harbored the glycosyltransferase gene *wbpL* (COG0472), which mediates synthesis of the *O*-specific antigen of LPS, but this gene was not present in the non–ROS-enhancing strains (Fig. 5 and S8, Table S2). In addition, gene cluster analysis using the cblaster tool revealed that both the ROS-enhancing and non–ROS-enhancing strains differed greatly in terms of the structure of the gene cluster responsible for synthesis of the *O*-specific antigen of LPS (Fig. 6). The *O*-specific antigen is reportedly involved in the virulence of plant-pathogenic *Pseudomonas* strains [36]. Further investigations will therefore focus on gene deletion analysis. On the other hand, although the extracellular part (growth medium) of most of the Gram-negative bacteria did not trigger cryptogein-induced ROS production (Table 1), we cannot rule out the possibility that during interaction with plant cells, these bacteria might secrete some ROS-enhancing components.

With regard to gram-positive bacteria, the cells of *Paenarthrobacter* sp. BR3S-9 and *Bacillus* sp. BR2S-4 exhibited ROS-enhancing activity, but the extracellular components did not (Table 1 and Fig. S6), suggesting that cell envelope–associated components play a role in the ROS-enhancing activity of these strains as well. In contrast, in 4 of the 6 gram-positive strains (*Arthrobacter* sp. BR2S-6, *Bacillus* sp. BR2R-4, *Microbacterium* sp. RS2P-3, and *Rhodococcus* sp. RS1R-6), extracellular components were found to enhance ROS production. The components produced by *Arthrobacter* sp. BR2S-6, *Bacillus* sp. BR2R-4, and *Microbacterium* sp. RS2P-3 were heat labile (Table 1), suggesting they could be proteins or peptides. Other studies have reported that proteins isolated from *Bacillus* strains can elicit plant immune responses [49, 50]. In contrast, characterization of the ROS-enhancing component produced by *Rhodococcus* sp. RS1R-6 revealed that it is heat stable (Table 1). *Rhodococcus* strains generally produce a variety of secondary metabolites [51], and thus, it is possible that the ROS-enhancing component produced by the strain in this study is a secondary metabolite.

## Conclusion

An assay method based on elicitor-induced ROS production in cultured plant cells enabled the discovery of novel plant immunity–activating bacteria from *R. sativus* var. *hortensis*. Three strains that colonize the interior of *Arabidopsis* plants enhanced resistance to the bacterial pathogens *P. syringae* pv. *tomato* DC3000 and/or *P. carotovorum* subsp. *carotovorum* NBRC 14082. The results in this study also suggest that the bacterial components involved in the ROS-enhancing activity may differ markedly by genus and species, although larger number of bacterial strains need to be studied to confirm such theory. It is conceivable that bacteria of different genera and species evolved their own plant immunity–activating systems through exposure to the plant environment. Furthermore, our comparative genomic analysis demonstrated that the structure of LPS in the outer cell envelope may play an important role in the ROS-enhancing activity of gram-negative *Pseudomonas* strains.

## Materials and methods

### Isolation and identification of bacteria from the interior of ***R. sativus var. hortensis***

*Raphanus sativus var. hortensis* plants were grown organically without the use of pesticides at the Suzuki Farm (Tachikawa, Tokyo, Japan) and collected in June 2019. Microorganisms were isolated from petioles and roots of the plants (Fig. S1) according to previous reports [22, 27], with some modifications: the fragments of petioles and roots were surface-sterilized by dipping in 1% sodium hypochlorite for 5 min, followed by immersion in 70% ethanol for 3–5 min. After rinsed with sterile water, each fragment was further cut and placed onto NBRC802 or ISP2 agar medium [27] and incubated at 30 °C for approximately 1 month. Taxonomic identification of the isolated bacteria was performed based on 16 S rRNA gene sequencing as reported previously [27, 52]. As the sequences of RS1R-3 and RS1R-4 were not successfully read using the primer 9 F [27], we used the primer 290 F (5′-CTGGTCTGAGAGGATGA-3′) instead.

### Measurement of cryptogein-induced ROS production in BY-2 cells after co-incubation with isolated bacteria

Cryptogein-induced ROS production was measured as reported previously [27]. In brief, the solution containing microbial cells and extracellular components (0.1 mL) was added to BY-2 cell suspension (60 g wet cell weight/L, 1.8 mL) in a well (3 mL) of a 6-well plate (Fig. S2). The mixture was incubated at room temperature on a rotary shaker (120 rpm) for 4 h. The cells were then collected by centrifugation (1000 rpm, 3 min) and suspended in fresh buffer to remove metabolites derived from microbial cells and BY-2 cells (e.g., organic compounds, ROS, and ROS scavengers). After addition of cryptogein (4–6 µM, 0.1 mL), the mixture was incubated at room temperature on a rotary shaker (120 rpm). ROS production induced by cryptogein was measured using a chemiluminescence assay with luminol. Samples that exhibited a relative chemiluminescence intensity more than 1.5 times that of mock-treated samples were selected as positives (Fig. 2 and S3). BY-2 cells preserved in our laboratory were used [27, 28].

### Treatment of whole ***Arabidopsis*** plants with isolated bacteria

Whole *Arabidopsis* plants were treated with isolated bacteria as reported previously [27, 53, 54]. In brief, whole plants of *Arabidopsis thaliana* Columbia-0 were inoculated with each strain of isolated bacteria by immersing the root tip of 7-day-old seedlings in diluted bacterial cell culture solution (OD_600_, 0.002) for 1 s. After inoculation, the plants were transferred to fresh 1/2 MS agar medium [27] and further cultivated in the growth chamber for 7 days. Seeds of *A. thaliana* Columbia-0 were obtained from The *Arabidopsis* Information Resource.

Resistance of isolated bacteria-colonized *Arabidopsis* plants to bacterial pathogens was evaluated using *P. syringae* pv. *tomato* DC3000 [53] and *P. carotovorum* subsp. *carotovorum* NBRC 14082 [55] as reported previously [27, 54]. In brief, pathogenic bacterial cell suspension (4 × 10^5^ CFU/mL; 40 mL) was dispensed into 1/2 MS agar medium containing 14-day-old *Arabidopsis* seedlings. After the plates were incubated at room temperature for 2 min, the cell suspension was decanted, and the seedlings on the plates were rinsed with sterile water. The plates were then incubated in a growth chamber with a light intensity of 150–200 µE m^− 2^ s^− 1^ (16 h light/8 h dark) and temperature of 22 °C. Plant disease symptoms were observed at 4 days after infection.

### Characterization of components enhancing cryptogein-induced ROS production

The bacterial cell culture solution was adjusted to an OD_600_ value of 0.8 using NBRC802 or ISP2 medium. To evaluate the thermal stability of the components, the bacterial cell culture solution was autoclaved. In contrast, to investigate the localization of the components, the bacterial cell culture solution was divided into cells and extracellular components by centrifugation (15,000 rpm, 10 min). The supernatant was collected and used as extracellular components. The precipitated cells were suspended in the same volume of NBRC802 or ISP2 medium (OD_600_, 0.8). After the solutions were diluted by a factor of 10 using ROS assay buffer, they were subjected to the measurement of cryptogein-induced ROS production, as described above.

### Comparative genomic analysis

Genome sequences of *Pseudomonas* strains BR1R-3, BR1R-5, RS1P-1, RS3R-1, and RS3R-2 were determined in our previous study [35]. In brief, for short-read sequencing, genomic libraries were prepared using a MGIEasy FS DNA Library Prep Set (MGI, Shenzhen, China), and sequencing was performed using a DNBSEQ-G400FAST sequencer and DNBSEQ-G400RS high-throughput rapid sequencing set (2 × 150 bp; MGI). The reads were utilized for de novo assembly using Platanus_B v1.3.2. Assembled genomes of *Pseudomonas* strains BR1R-3 (accession no. BSCL00000000), BR1R-5 (BSCO00000000), RS1P-1 (BSCP00000000), RS3R-1 (BSCQ00000000), and RS3R-2 (BSCR00000000) [35] and reference genomes of *P. alcaliphila* NBRC 102411 (accession no. BCZV00000000), *P. oleovorans* NBRC 13583 (BDAL00000000), and *P. oleovorans* NBRC 14167 (BDAJ00000000) were used for the comparative genomic analysis. Clusters of orthologous genes from these *Pseudomonas* genomes were listed using SonicParanoid with default parameter settings [56]. Subsequently, the list was filtered by the clusters present in all strains that enhanced cryptogein-induced ROS production in BY-2 cells (BR1R-3, BR1R-5, RS1P-1, and RS3R-1) but absent in all non–ROS-enhancing strains (RS3R-2, NBRC 13583, NBRC 14167, and NBRC 102411) [57]. Proteins were assigned to the clusters of orthologous genes using EggNOG-mapper v2.1.3 [58] with default parameters, based on the EggNOG 5.0 database [59]. A heatmap was created using TBtools v1.0986853 [60]. Genome sequences were searched for *O*-specific antigen gene clusters with the *wbpL* gene and its related genes of *P. aeruginosa* PAO1 [36] as a query using cblaster v1.3.8 [61] according to our previous report [62] with some modifications. Gene cluster comparison was visualized using clinker [63].

## Electronic supplementary material

Below is the link to the electronic supplementary material.

Supplementary Material 1

## Data Availability

Data regarding the genomes of *Pseudomonas* strains BR1R-3, BR1R-5, RS1P-1, RS3R-1, and RS3R-2 were submitted to the NCBI GenBank and are publicly available under BioProject accession numbers PRJDB14730 and PRJDB14766. All other data generated during this study are included in this published article (and its Supplementary Information files).

## Abbreviations

CFU: Colony forming unit
ISR: Induced systemic resistance
LPS: Lipopolysaccharide
ROS: Reactive oxygen species
