# Supplementary material for: Diversity and characteristics of plant immunity–activating bacteria from Brassicaceae plants
Source: BMC Microbiol. 2023 Jul 5;23:175. doi: 10.1186/s12866-023-02920-y (PMC10320978; doi:10.1186/s12866-023-02920-y)
Supplement: Supplementary file 1 — Supplementary Material 1 [file 12866_2023_2920_MOESM1_ESM.docx]

Supplementary Information

**Diversity and characteristics of plant immunity–activating bacteria from *Brassicaceae* plants**

Hiroki Kaneko, Fuma Miyata, Mari Kurokawa, Kenji Hashimoto, Kazuyuki Kuchitsu, Toshiki Furuya*

----------------------------------------------------------------------------------------------------------

Department of Applied Biological Science, Faculty of Science and Technology, Tokyo University of Science, 2641, Yamazaki, Noda, Chiba 278-8510, Japan

*Correspondence: tfuruya@rs.tus.ac.jp

**Table S1.** Bacterial strains recovered from the interior of *R. sativus var. hortensis*.

| Strain | Host  tissue | Phylum | Accession no. | Closest type strain  (accession no.) | Sequence  length (bp) | Similarity (%) |
| --- | --- | --- | --- | --- | --- | --- |
| RS1P-1 | Petiole | *Proteobacteria* | LC646120 | *Pseudomonas fulva* NBRC 16637T  (NR_113857.1) | 708 | 99 |
| RS1P-2 | Petiole | *Proteobacteria* | LC646121 | *Agrobacterium tumefaciens* NBRC 12607  (AB680300.1) | 559 | 99 |
| RS1P-4 | Petiole | *Proteobacteria* | LC646122 | *Agrobacterium tumefaciens* NBRC 12607  (AB680300.1) | 799 | 100 |
| RS1P-5 | Petiole | *Proteobacteria* | LC646123 | *Pseudomonas fulva* NBRC 16637T  (NR_113857.1) | 646 | 99 |
| RS1P-6 | Petiole | *Proteobacteria* | LC646124 | *Pseudomonas fulva* NBRC 16637T  (NR_113857.1) | 421 | 99 |
| RS1R-1 | Root | *Proteobacteria* | LC646131 | *Pseudomonas viridiflava* DSM 6694T  (NR_114482.1) | 427 | 99 |
| RS1R-2 | Root | *Proteobacteria* | LC646132 | *Pseudomonas fulva* NBRC 16637T  (NR_042351.1) | 479 | 99 |
| RS1R-3 | Root | *Proteobacteria* | LC646133 | *Pantoea brenneri* LMG 5343T  (NR_116748.1) | 515 | 99 |
| RS1R-4 | Root | *Proteobacteria* | LC646134 | *Pantoea brenneri* LMG 5343T  (NR_116748.1) | 258 | 98 |
| RS1R-5 | Root | *Proteobacteria* | LC646135 | *Rhizobium indigoferae* NBRC 100398T  (NR_113895.1) | 409 | 100 |
| RS1R-6 | Root | *Actinobacteria* | LC646136 | *Rhodococcus qingshengii* JCM 15477T  (NR_043535.1) | 854 | 99 |
| RS2P-1 | Petiole | *Proteobacteria* | LC646125 | *Xanthomonas campestris* ATCC 33913T  (NR_074936.1) | 411 | 97 |
| RS2P-2 | Petiole | *Proteobacteria* | LC646126 | *Pseudomonas fulva* NBRC 16637T  (NR_042351.1) | 567 | 99 |
| RS2P-3 | Petiole | *Actinobacteria* | LC646127 | *Microbacterium maritypicum* DSM 12512T  (NR_114986.1) | 665 | 99 |
| RS2P-4 | Petiole | *Proteobacteria* | LC646128 | *Xanthomonas campestris* ATCC 33913T  (NR_074936.1) | 455 | 97 |
| RS2P-6 | Petiole | *Proteobacteria* | LC646129 | *Pseudomonas tolaasii* ATCC 33618T  (NR_114481.1) | 543 | 97 |
| RS2P-7 | Petiole | *Proteobacteria* | LC646130 | *Agrobacterium tumefaciens* NBRC 12607  (AB680300.1) | 467 | 99 |
| RS2R-1 | Root | *Proteobacteria* | LC646137 | *Enterobacter mori* LMG 25706T  (MG846019.1) | 614 | 99 |
| RS2R-3 | Root | *Proteobacteria* | LC646138 | *Enterobacter soli* ATCC BAA-2102T  (NR_117547.1) | 571 | 99 |
| RS3R-1 | Root | *Proteobacteria* | LC646139 | *Pseudomonas fulva* NBRC 16637T  (NR_042351.1) | 515 | 99 |
| RS3R-2 | Root | *Proteobacteria* | LC646140 | *Pseudomonas tolaasii* ATCC 33618T  (NR_114481.1) | 845 | 98 |
| RS3R-3 | Root | *Proteobacteria* | LC646141 | *Pseudomonas fulva* NBRC 16637T  (NR_042351.1) | 606 | 99 |
| RS3R-4 | Root | *Proteobacteria* | LC646142 | *Leclercia adecarboxylata* NBRC 102595T  (NR_114154.1) | 833 | 100 |
| RS3R-6 | Root | *Actinobacteria* | LC646143 | *Pseudomonas fulva* NBRC 16637T  (NR_042351.1) | 620 | 99 |
| RS3R-7 | Root | *Actinobacteria* | LC646144 | *Plantibacter flavus* DSM 14012T  (AJ310417.1) | 408 | 99 |

**Table S2.** List of clusters of orthologous genes present in all ROS-enhancing strains that were absent in all non–ROS-enhancing strains.

| group_id | COG  category | Description |
| --- | --- | --- |
| COG1529 | C | Cytochrome c |
| COG2080 | C | 2Fe-2S -binding domain protein |
| COG0604 | C | PFAM Alcohol dehydrogenase zinc-binding domain protein |
| COG0247 | C | FAD linked oxidase domain protein |
| COG0604 | C | Zinc-binding dehydrogenase |
| COG1301 | C | Responsible for the transport of dicarboxylates such as succinate, fumarate, and malate |
| COG0446 | C | PFAM FAD-dependent pyridine nucleotide-disulphide oxidoreductase |
| COG2864 | C | formate dehydrogenase |
| COG0243 | C | Belongs to the prokaryotic molybdopterin-containing oxidoreductase family |
| COG4657 | C | Part of a membrane complex involved in electron transport |
| COG0403 | E | The glycine cleavage system catalyzes the degradation of glycine. The P protein binds the alpha-amino group of glycine through its pyridoxal phosphate cofactor |
| COG0436 | E | Cys/Met metabolism PLP-dependent enzyme |
| COG1280 | E | Lysine transporter LysE |
| COG0346 | E | PFAM Glyoxalase bleomycin resistance protein dioxygenase |
| COG1231 | E | amine oxidase |
| COG0477 | E, G, P | PFAM major facilitator superfamily MFS_1 |
| COG0477 | E, G, P | Permeases of the major facilitator superfamily |
| COG0477 | E, G, P | PFAM General substrate transporter |
| COG0477 | E, G, P | PFAM major facilitator superfamily MFS_1 |
| COG2233 | F | xanthine |
| COG0590 | F, J | CMP dCMP deaminase, zinc-binding |
| COG2271 | G | Major facilitator superfamily |
| COG2271 | G | Major facilitator superfamily |
| COG2271 | G | COG0477 Permeases of the major facilitator superfamily |
| COG3232 | G | 5-carboxymethyl-2-hydroxymuconate isomerase |
| COG3958 | G | Transketolase |
| COG3959 | G | Transketolase |
| COG0451 | G, M | NAD(P)H-binding |
| COG0642 | H | His Kinase A (phosphoacceptor) domain |
| COG1903 | H | Catalyzes the methylation of C-1 in cobalt-precorrin-5B to form cobalt-precorrin-6A |
| COG0657 | I | Alpha beta hydrolase |
| COG3255 | I | transcription factor jumonji |
| COG4925 | I | sulfurtransferase activity |
| COG1028 | I, Q | Dehydrogenases with different specificities (Related to short-chain alcohol dehydrogenases) |
| COG1234 | J | Zinc phosphodiesterase, which displays some tRNA 3'- processing endonuclease activity. Probably involved in tRNA maturation, by removing a 3'-trailer from precursor tRNA |
| COG1514 | J | Hydrolyzes RNA 2',3'-cyclic phosphodiester to an RNA 2'- phosphomonoester |
| COG1670 | J | Siderophore biosynthesis protein |
| COG1921 | J | Converts seryl-tRNA(Sec) to selenocysteinyl-tRNA(Sec) required for selenoprotein biosynthesis |
| COG1208 | J, M | Glucose-1-phosphate cytidylyltransferase |
| COG0583 | K | Transcriptional regulator |
| COG1396 | K | Transcriptional regulator |
| COG0583 | K | Transcriptional regulator |
| COG1167 | K | Aminotransferase, class I |
| COG0454 | K | Acetyltransferase (GNAT) domain |
| COG1595 | K | Sigma-70, region 4 |
| COG0583 | K | Transcriptional regulator |
| COG0317 | K, T | HD domain |
| COG3568 | L | PFAM Endonuclease exonuclease phosphatase |
| COG0708 | L | Exodeoxyribonuclease III |
| COG0122 | L | DNA-3-methyladenine glycosylase |
| COG0789 | L | Interacts with CbpA and inhibits both the DnaJ-like co- chaperone activity and the DNA binding activity of CbpA. Together with CbpA, modulates the activity of the DnaK chaperone system. Does not inhibit the co-chaperone activity of DnaJ |
| COG1201 | L | DEAD/H associated |
| COG0412 | M | dienelactone hydrolase |
| COG0472 | M | Catalyzes the transfer of the GlcNAc-1-phosphate moiety from UDP-GlcNAc onto the carrier lipid undecaprenyl phosphate (C55-P), yielding GlcNAc-pyrophosphoryl-undecaprenyl (GlcNAc-PP- C55) |
| COG3773 | M | Cell wall hydrolase |
| COG1368 | M | Sulfatase |
| COG1216 | M | PFAM glycosyl transferase family 2 |
| COG1898 | M | Catalyzes the epimerization of the C3' and C5'positions of dTDP-6-deoxy-D-xylo-4-hexulose, forming dTDP-6-deoxy-L-lyxo-4- hexulose |
| COG0451 | M | CDP-glucose 4,6-dehydratase |
| COG4948 | M | Belongs to the mandelate racemase muconate lactonizing enzyme family |
| COG3264 | M | mechanosensitive ion channel |
| COG3659 | M | PFAM Carbohydrate-selective porin OprB |
| COG2373 | M, N, U | Bacterial Ig-like domain (group 3) |
| COG3170 | N, U | Tfp pilus assembly protein FimV |
| COG0265 | O | Trypsin-like serine proteases, typically periplasmic, contain C-terminal PDZ domain |
| COG3058 | O | Necessary for formate dehydrogenase activity |
| COG0600 | P | binding-protein-dependent transport systems inner membrane component |
| COG1116 | P | ABC transporter |
| COG0715 | P | NMT1-like family |
| COG0428 | P | Zinc iron permease |
| COG3119 | P | Arylsulfatase A and related enzymes |
| COG1914 | P | Natural resistance-associated macrophage protein |
| COG1457 | P | Belongs to the purine-cytosine permease (2.A.39) family |
| COG1020 | Q | Non-ribosomal peptide synthetase modules and related proteins |
| COG5301 | S | Phage tail-collar fibre protein |
| COG4104 | S | Uncharacterized alpha/beta hydrolase domain (DUF2235) |
| COG0599 | S | Carboxymuconolactone decarboxylase family |
| COG2194 | S | membrane-associated metal-dependent hydrolase |
| COG3741 | S | CS1 type fimbrial major subunit |
| COG4125 | S | PFAM transmembrane pair domain protein |
| COG2259 | S | DoxX family |
| COG0393 | S | Belongs to the UPF0145 family |
| COG1881 | S | PFAM PEBP family protein |
| COG3011 | S | Protein conserved in bacteria |
| COG1917 | S | conserved protein, contains double-stranded beta-helix domain |
| COG3767 | S | EF hand |
| COG2604 | S | Motility accesory factor maf-2 |
| COG2910 | S | NAD(P)H-binding |
| COG4319 | S | ketosteroid isomerase |
| COG1289 | S | Fusaric acid resistance protein |
| COG2199 | T | (GGDEF) domain |
| COG2199 | T | Diguanylate cyclase |
| COG0664 | T | cyclic nucleotide-binding |
| COG0467 | T | Circadian clock protein KaiC |
| COG0745 | T | response regulator |
| COG4943 | T | PFAM EAL domain protein |
| COG5001 | T | Diguanylate cyclase |
| COG2199 | T | Diguanylate cyclase |
| COG0457 | T | Response regulator receiver |
| COG1132 | V | Involved in lipid A export and possibly also in glycerophospholipid export and for biogenesis of the outer membrane. Transmembrane domains (TMD) form a pore in the inner membrane and the ATP-binding domain (NBD) is responsible for energy generation |
| COG3510 | V | Cephalosporin hydroxylase |
| COG2274 | V | ABC-type bacteriocin lantibiotic exporters, contain an N-terminal double-glycine peptidase domain |


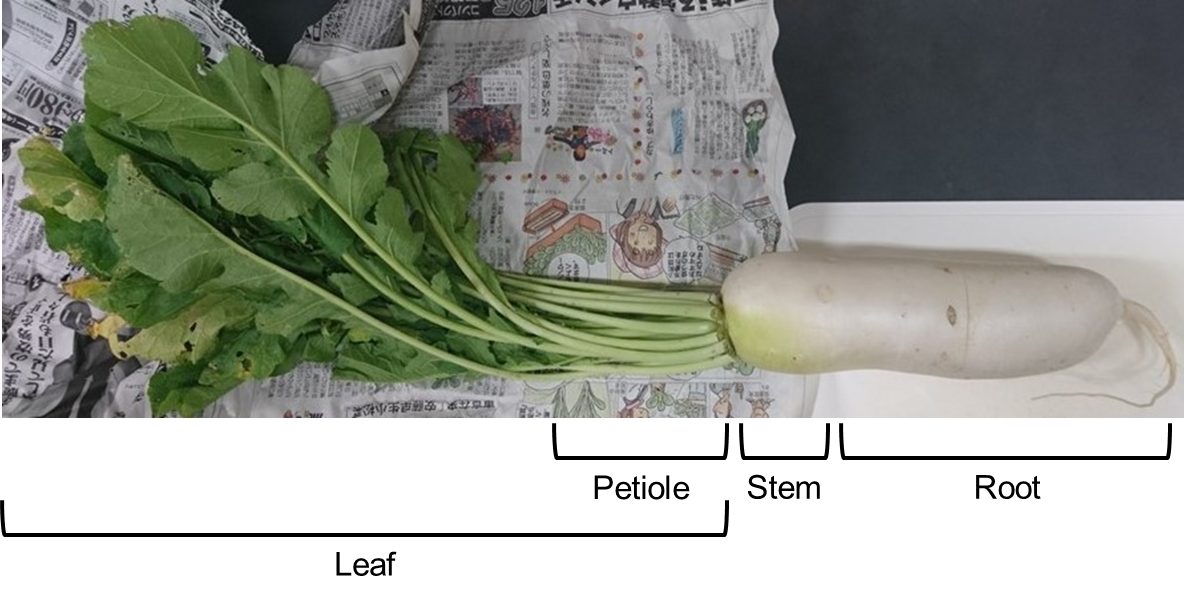


**Fig. S1** Photograph of *R. sativus* var. *hortensis*.


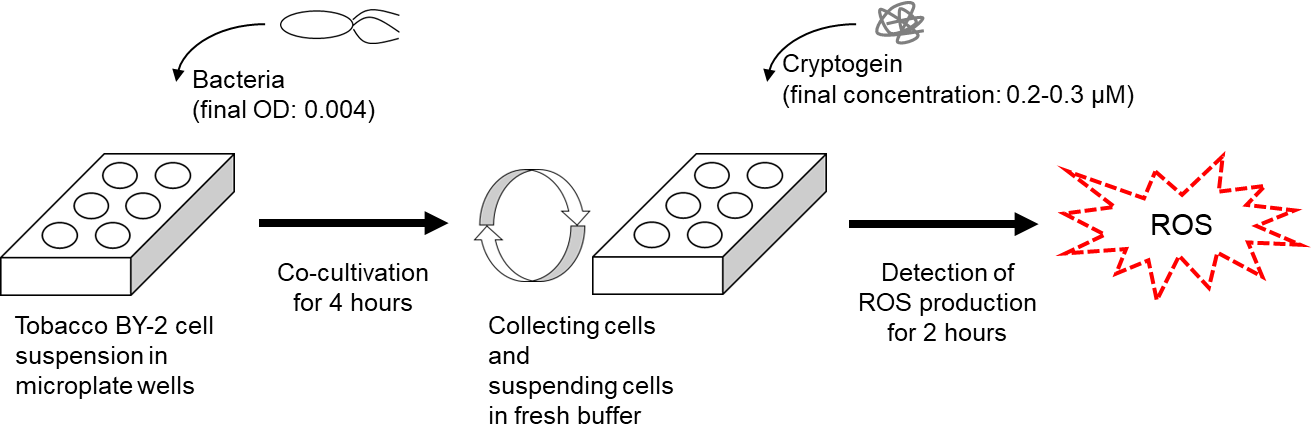


**Fig. S2** Schematic illustration of assays for cryptogein-induced ROS production in BY-2 cells co-incubated with bacteria.


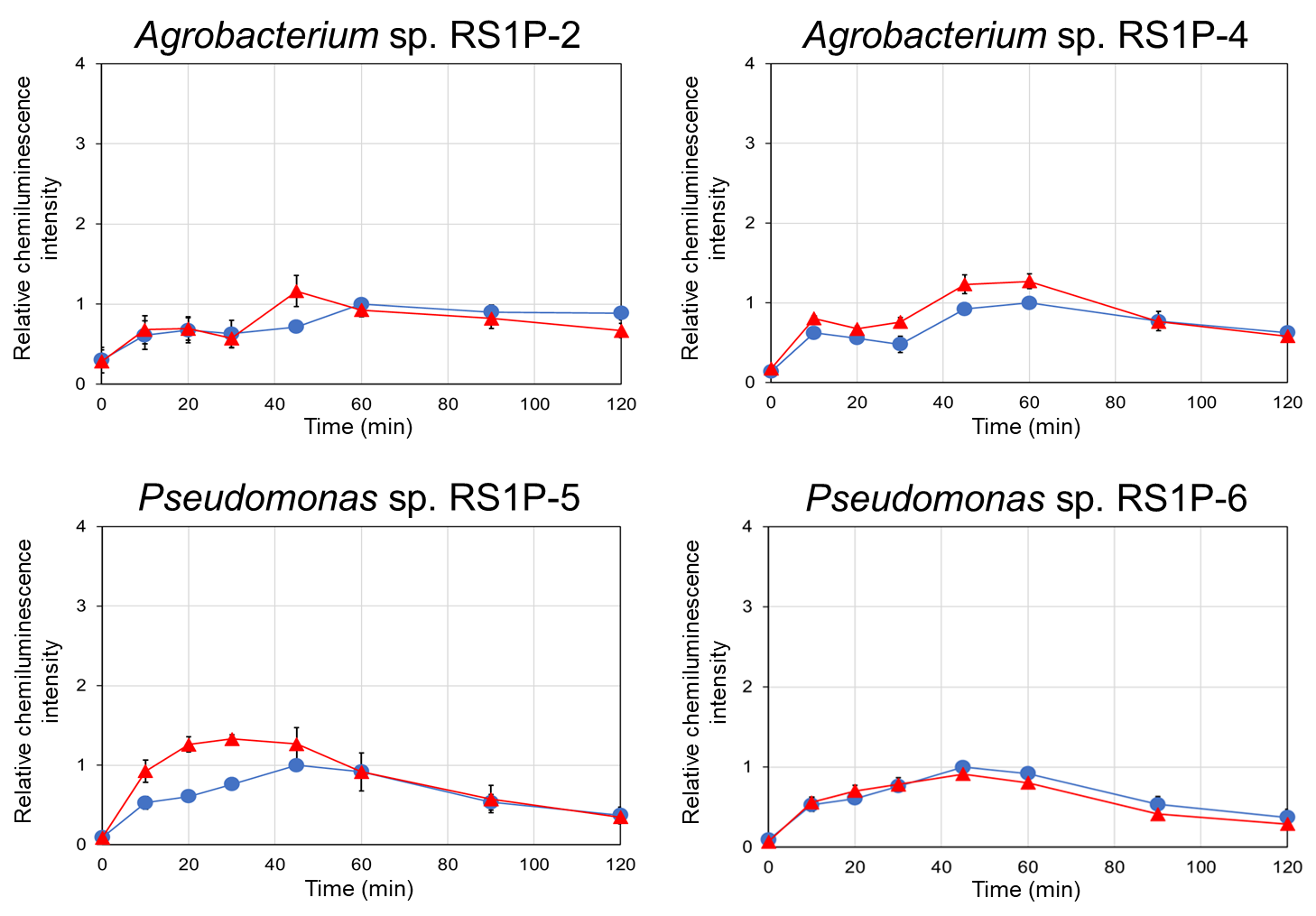


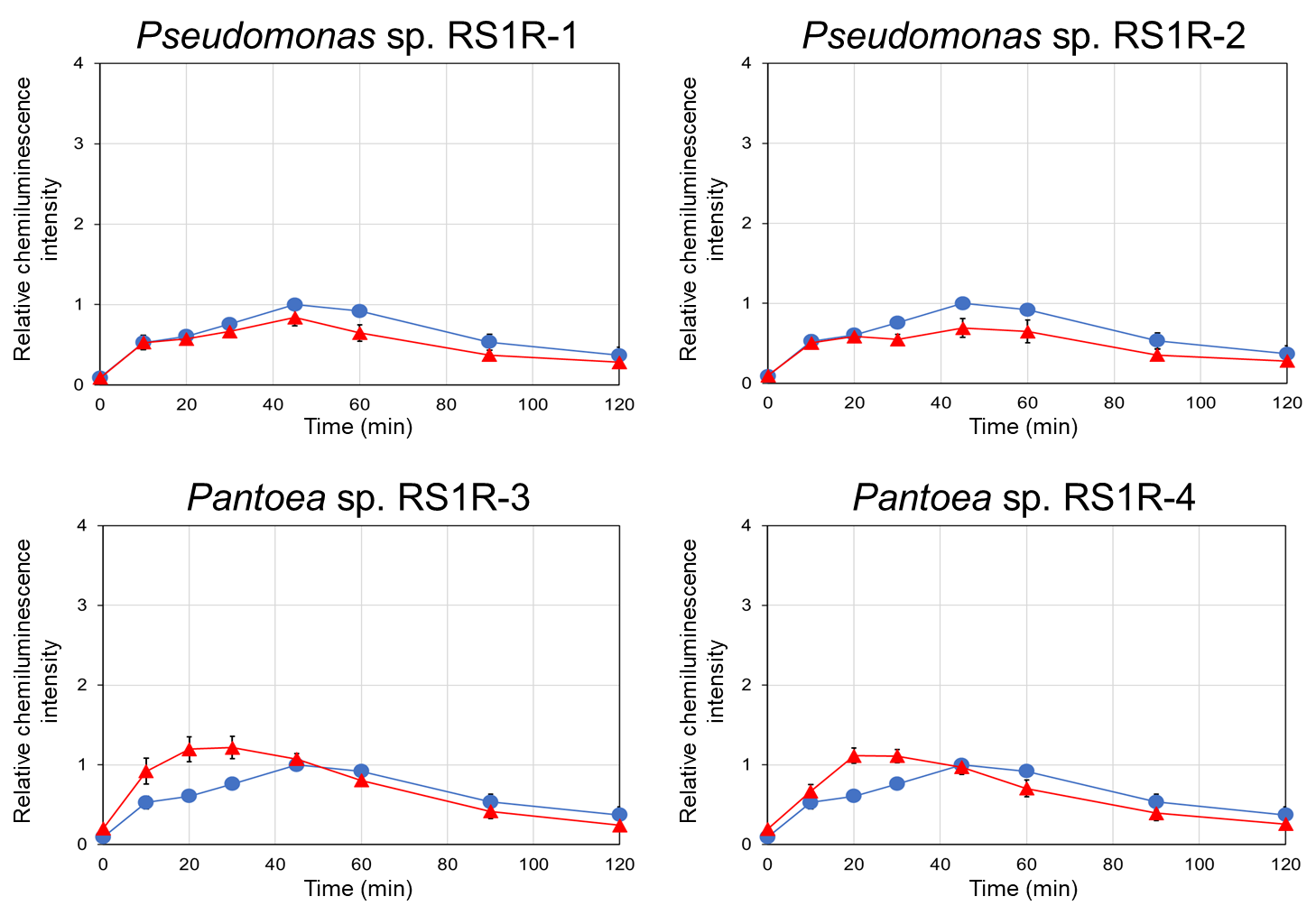


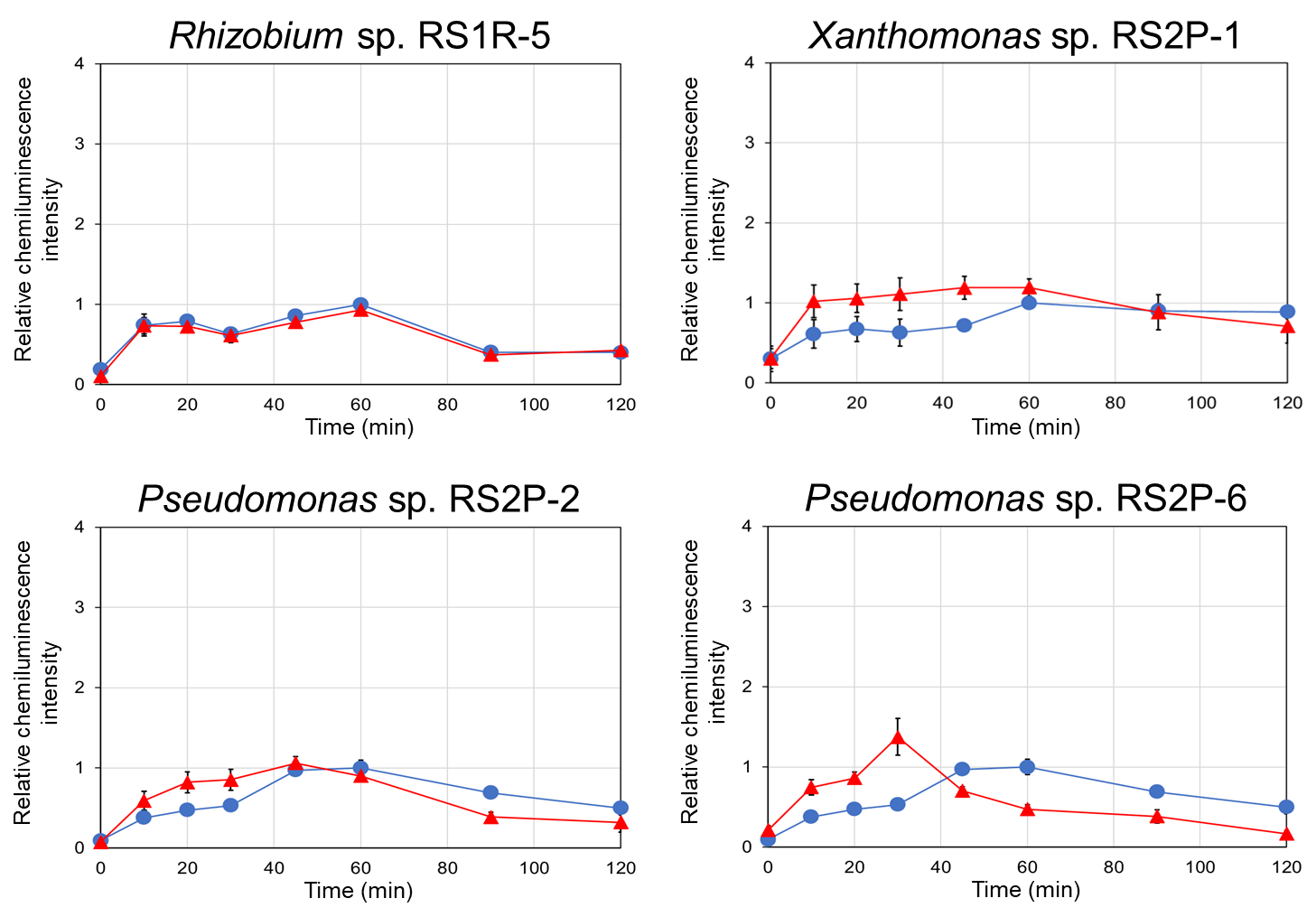


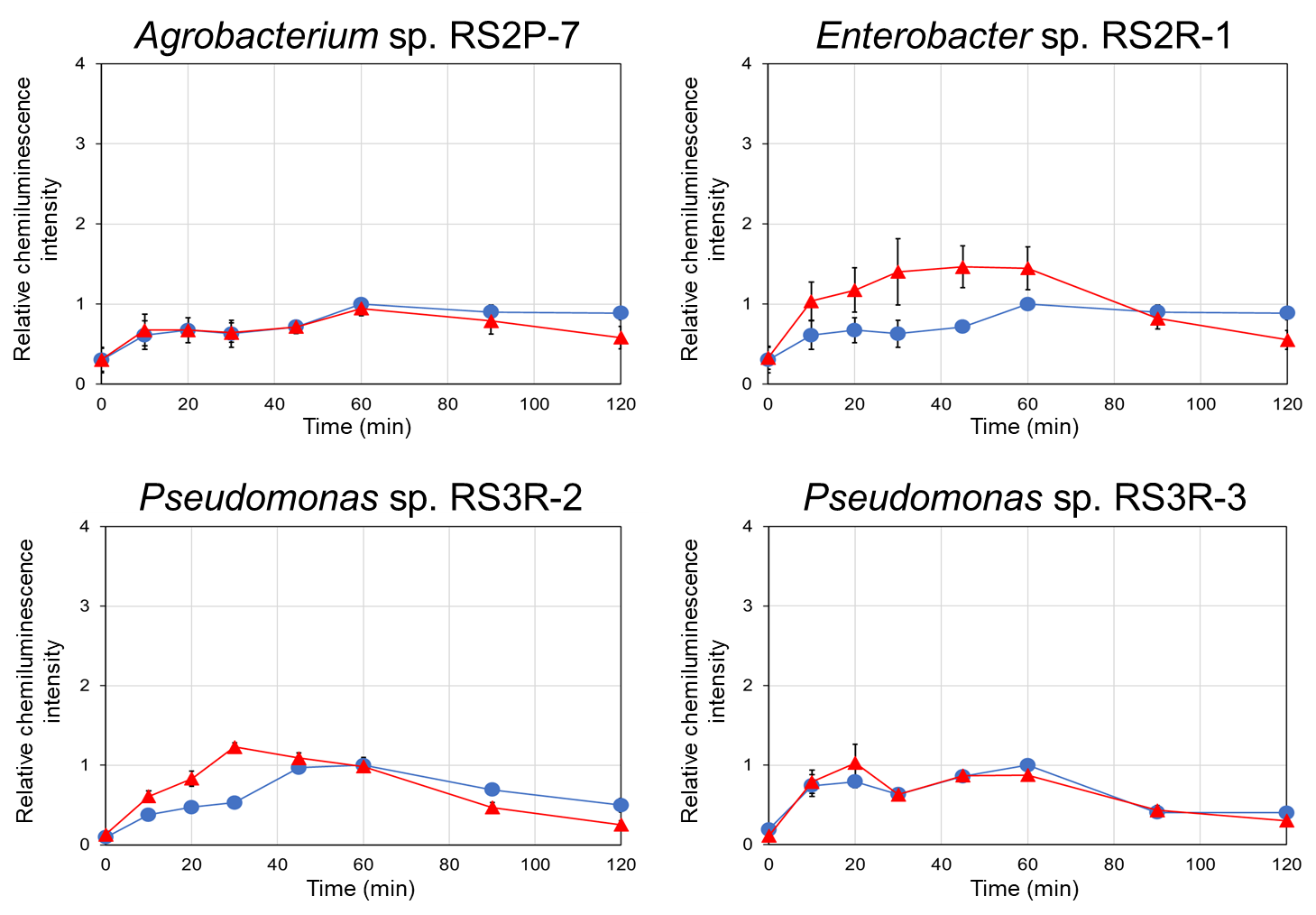


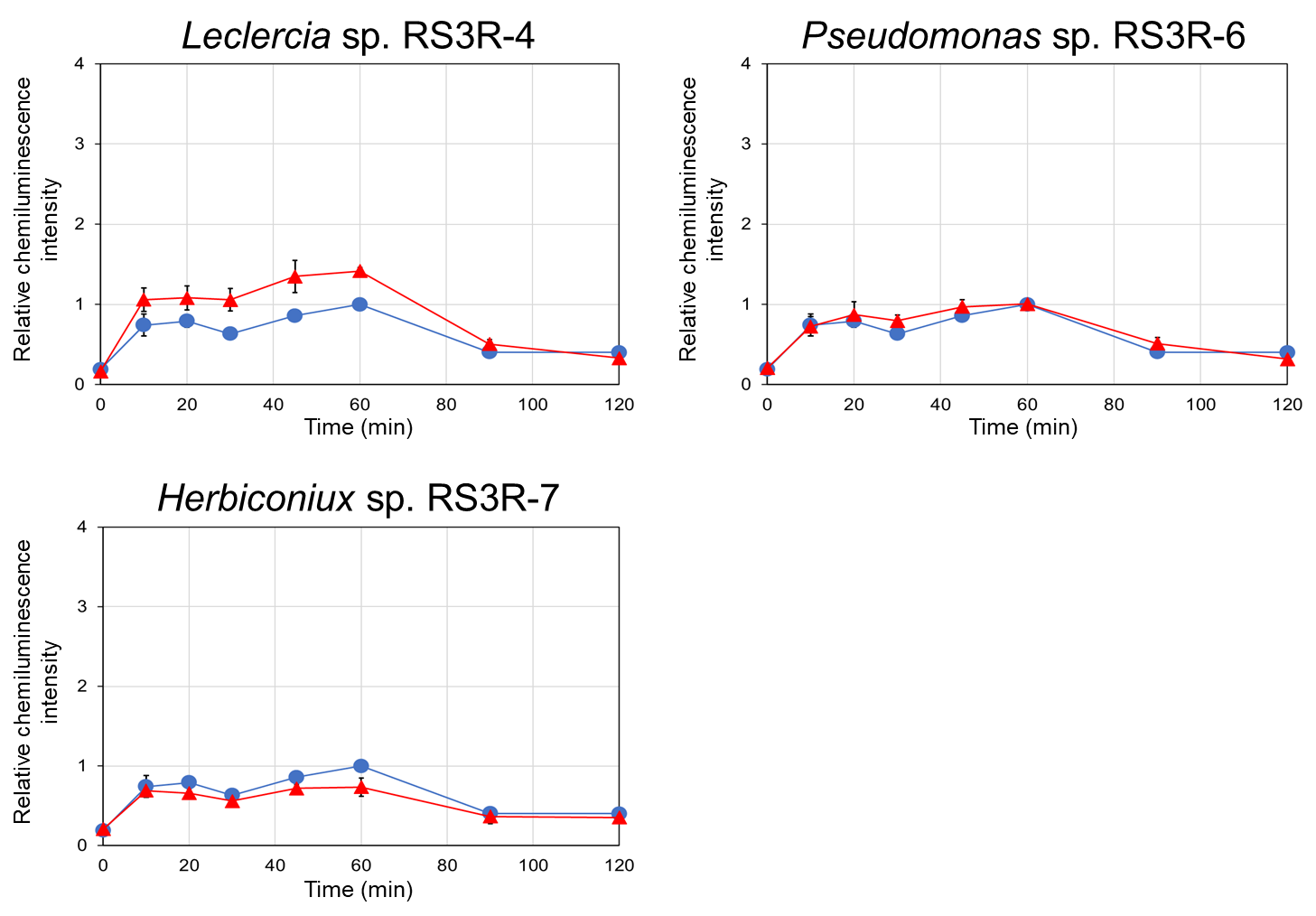


**Fig. S3** Cryptogein-induced ROS production in BY-2 cells co-incubated with bacteria. Bacteria that did not enhanced cryptogein-induced ROS production are shown. BY-2 cells were co-incubated with bacteria of each strain (∆) or subjected to mock treatment (only a mixture of medium and buffer, ○), and then cryptogein was added. ROS production was monitored based chemiluminescence. The maximum value of the mock control was expressed as 1.0, and relative chemiluminescence intensity is shown. Average values ± SE from three independent experiments are presented.


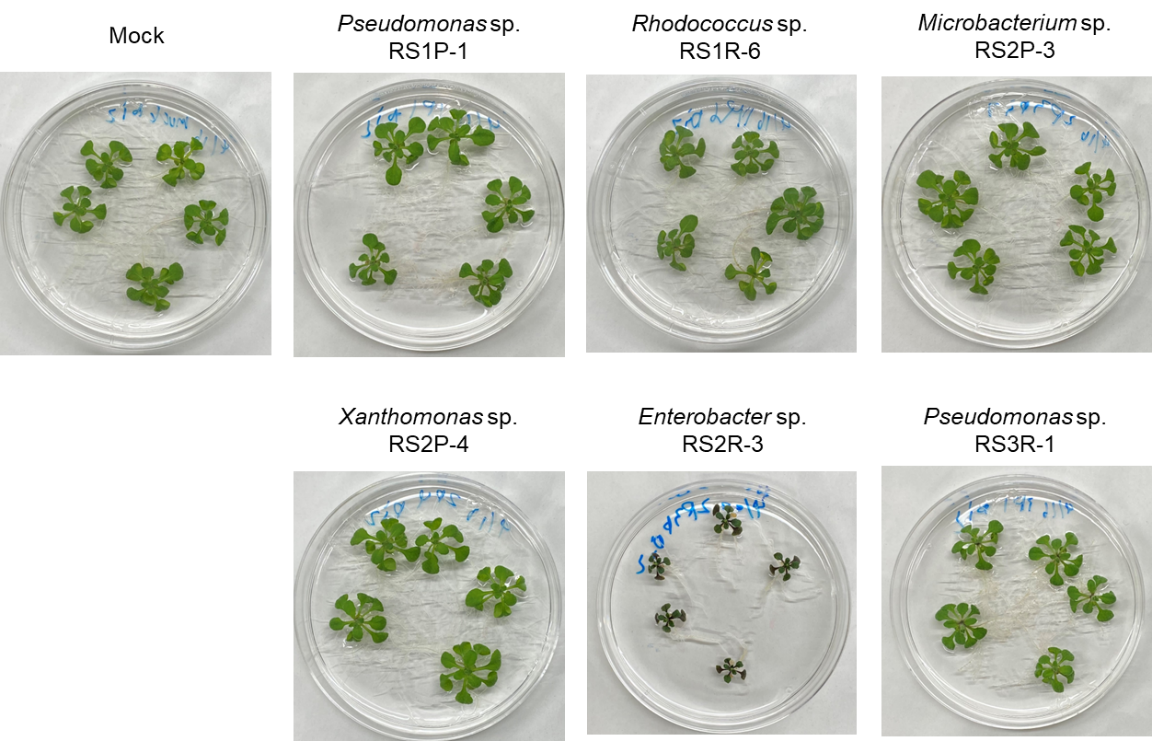


(a)


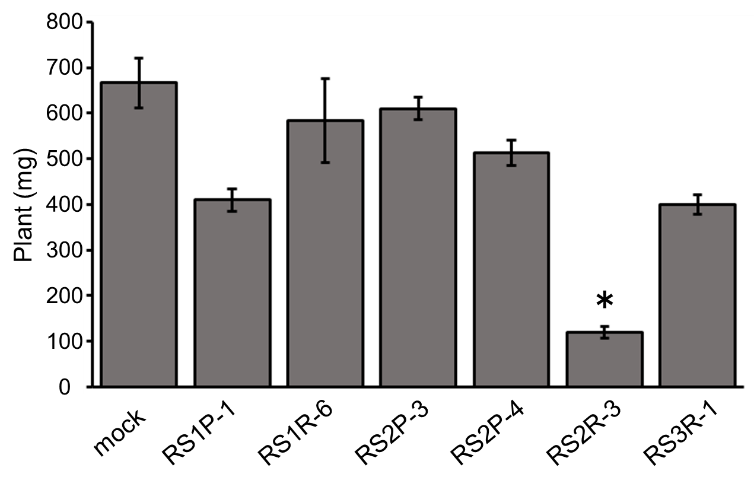


(b)

**Fig. S4** *Arabidopsis* plants inoculated with the selected bacteria. Plants were inoculated with each strain of selected bacteria or mock treatment (only the medium) by immersing the root tip of 7-day-old seedlings in bacterial cell culture solution, followed by cultivation for 7 days. (a), representative photographs. (b), fresh weight of a plant. Average values ± SE from three independent experiments are presented. Asterisks indicate a significant difference from the mock control based on Student’s *t*-test (*, *P*<0.05).


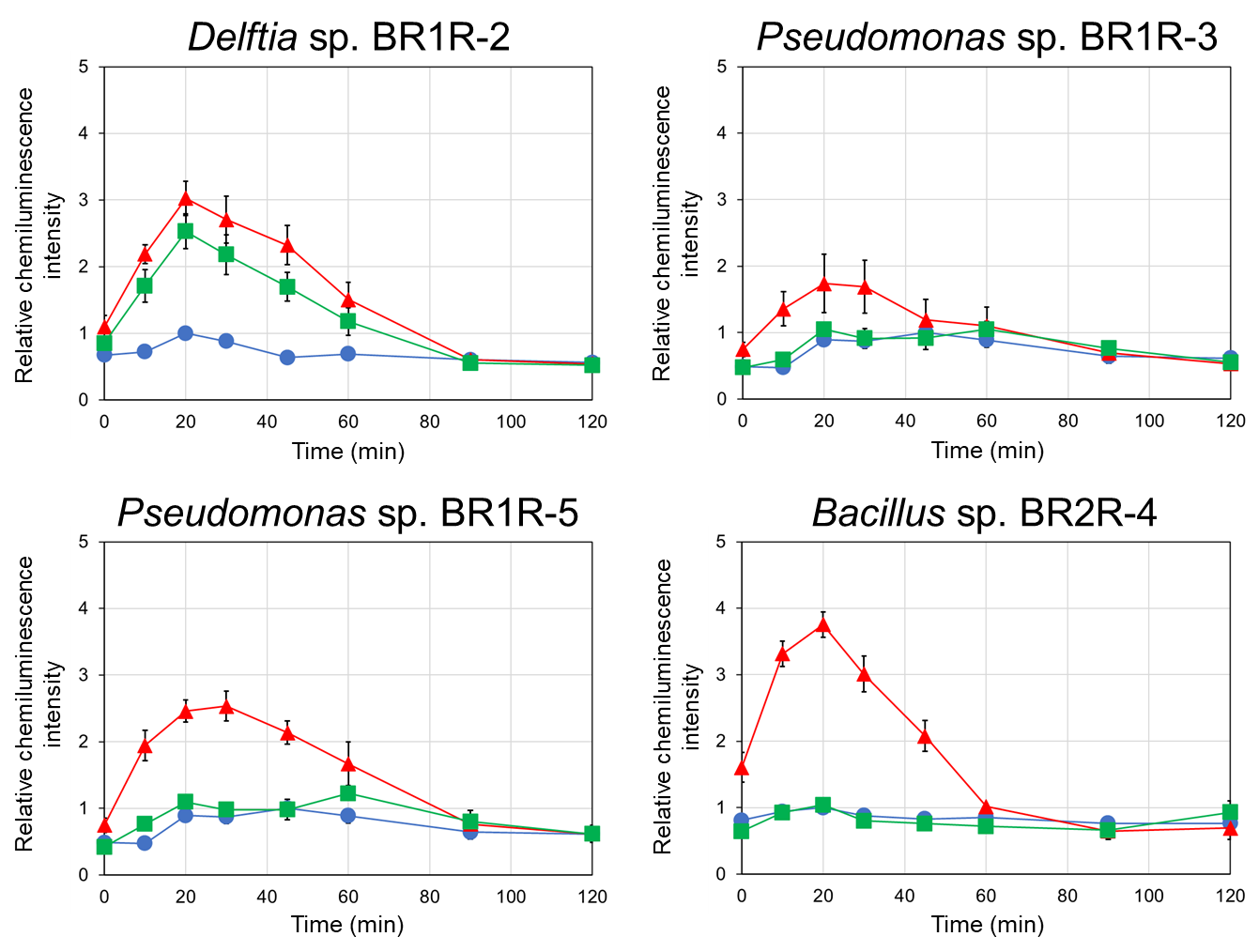


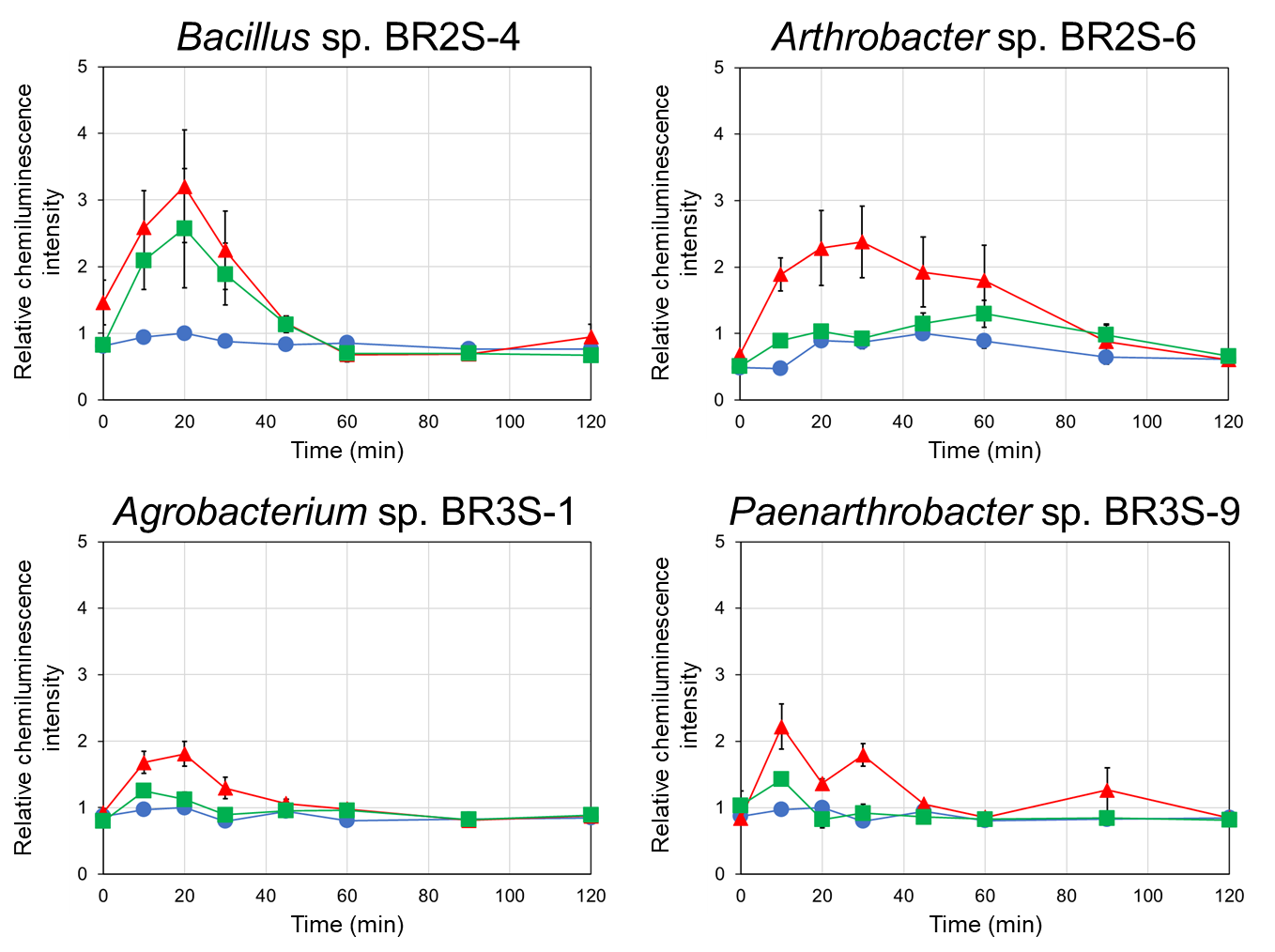


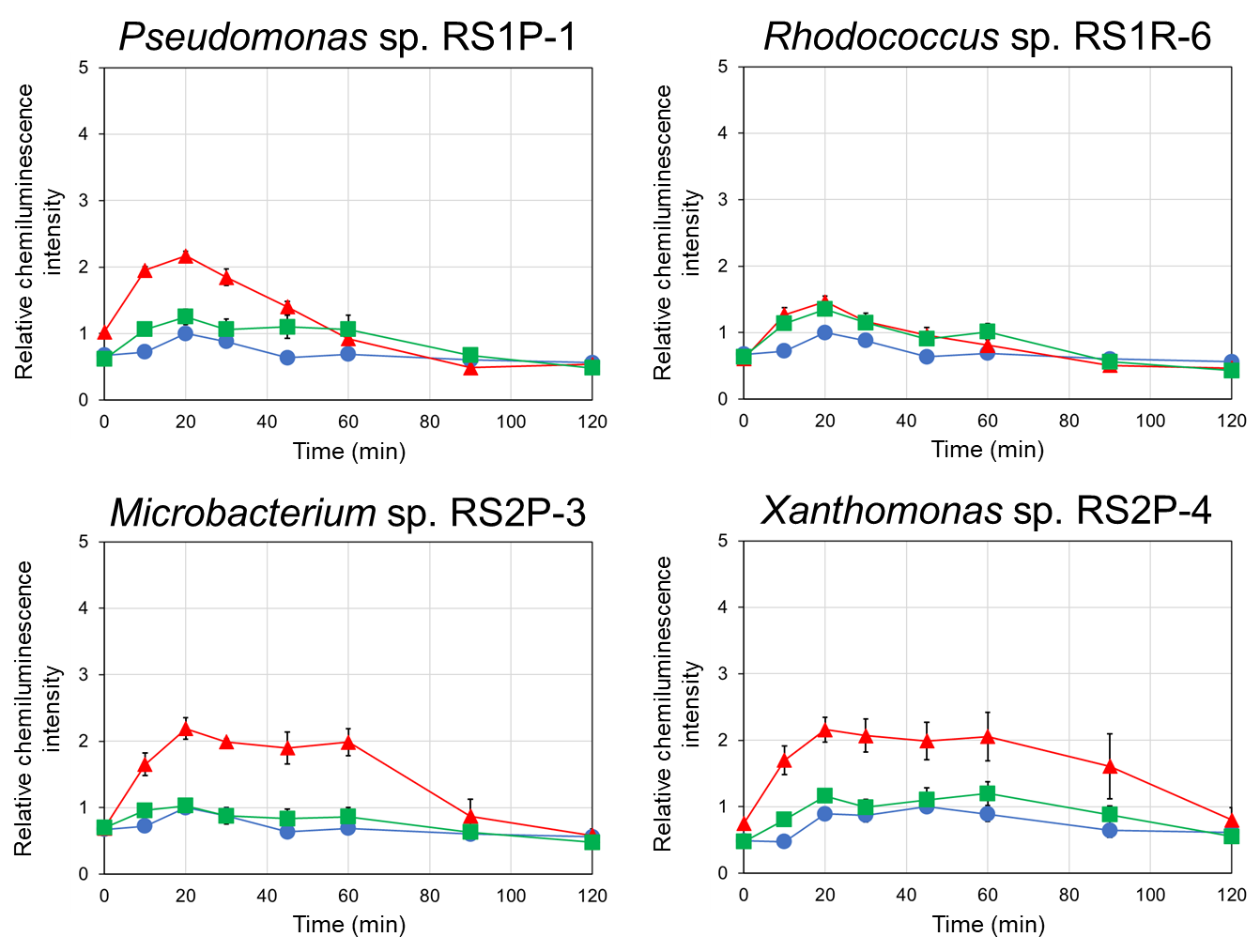


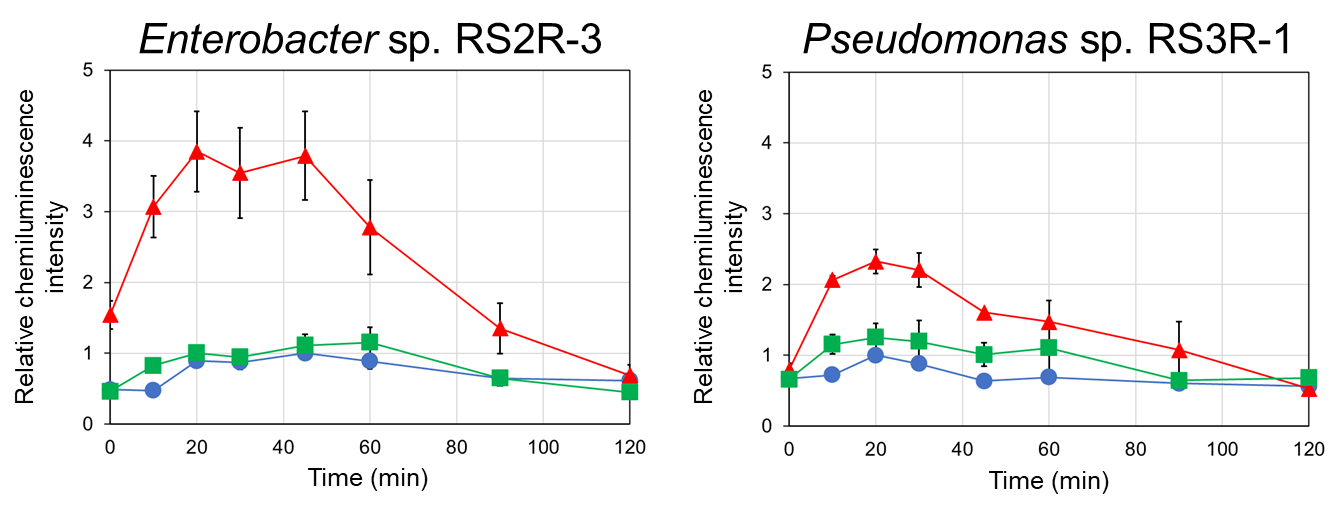


**Fig. S5** Thermal stability of components that enhanced cryptogein-induced ROS production. BY-2 cells were co-incubated with bacterial cell culture solution (∆) or the solution after autoclaving (□), or subjected to mock treatment (only a mixture of medium and buffer, ○), and then cryptogein was added. ROS production was monitored based on chemiluminescence. The maximum value of the mock control was expressed as 1.0, and relative chemiluminescence intensity is shown. Average values ± SE from three independent experiments are presented.


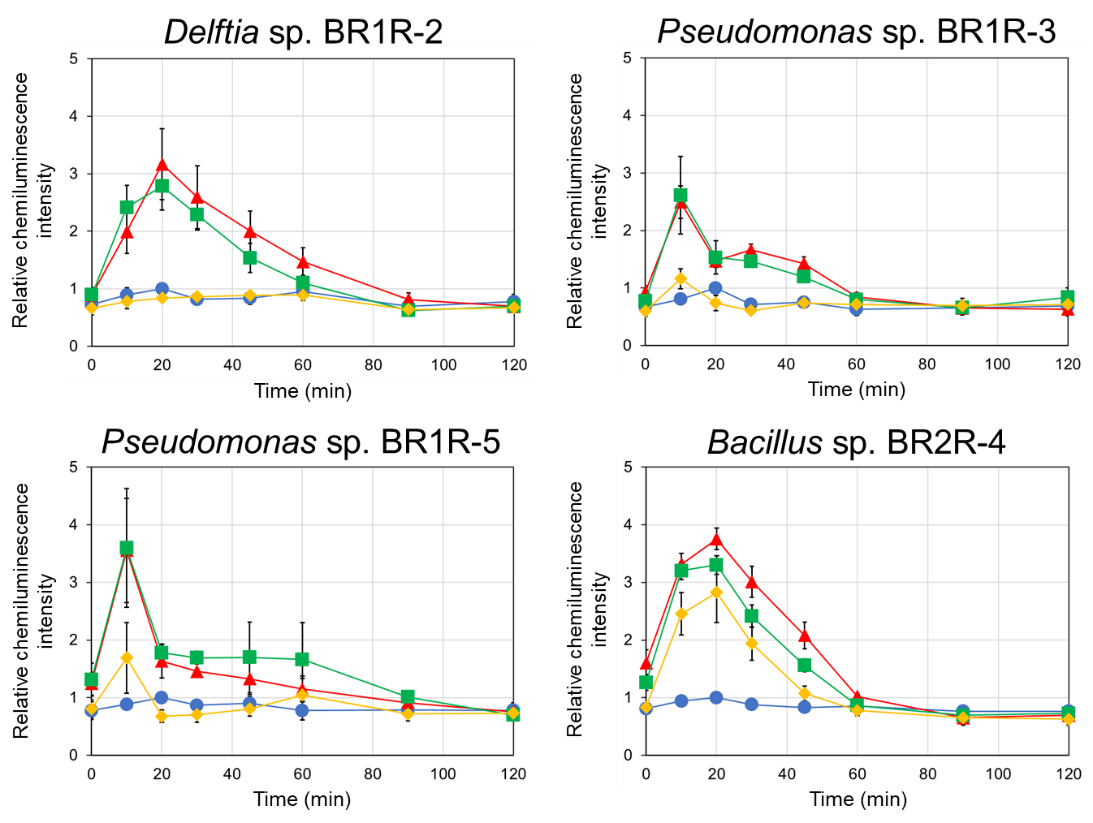


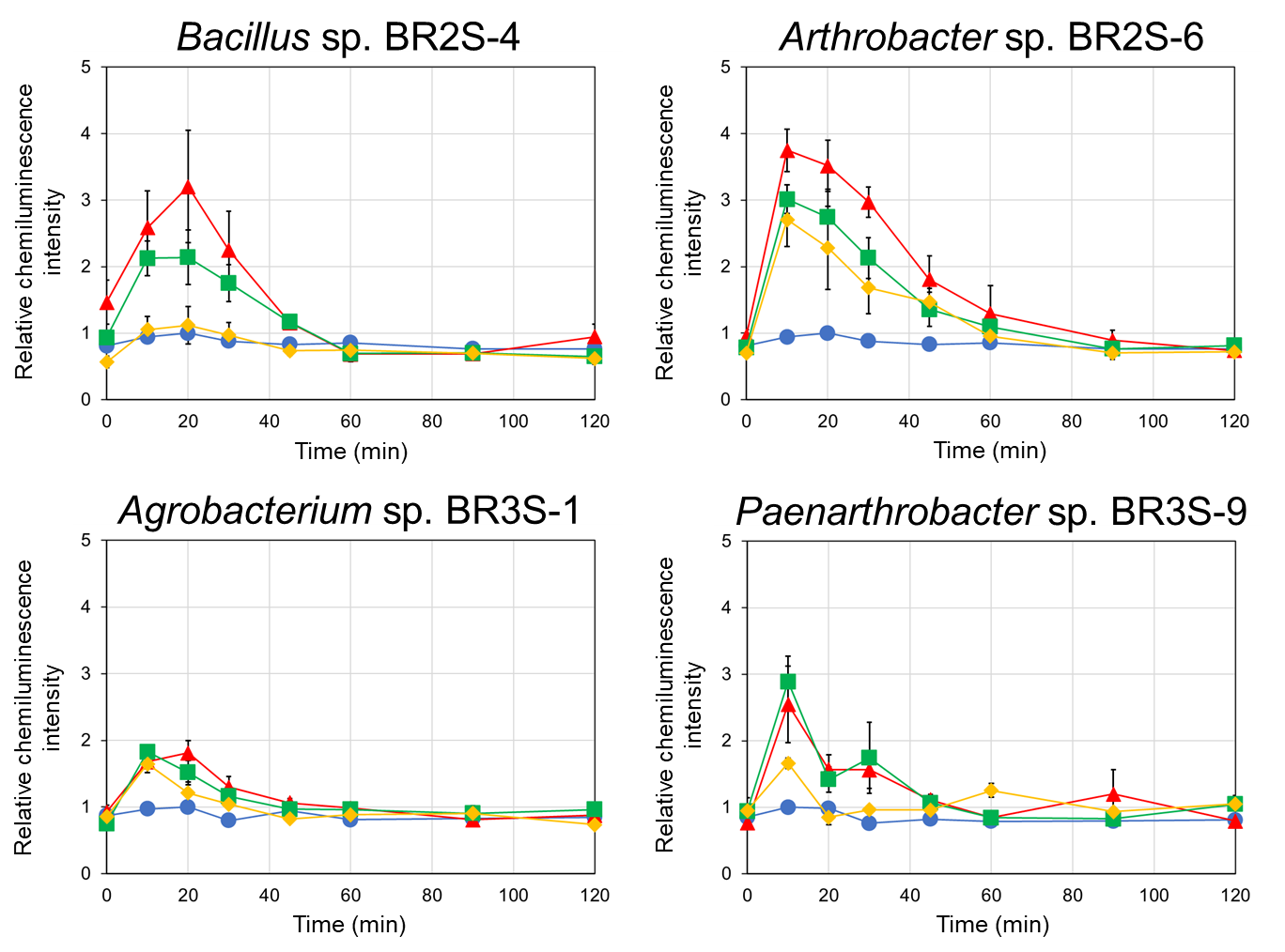


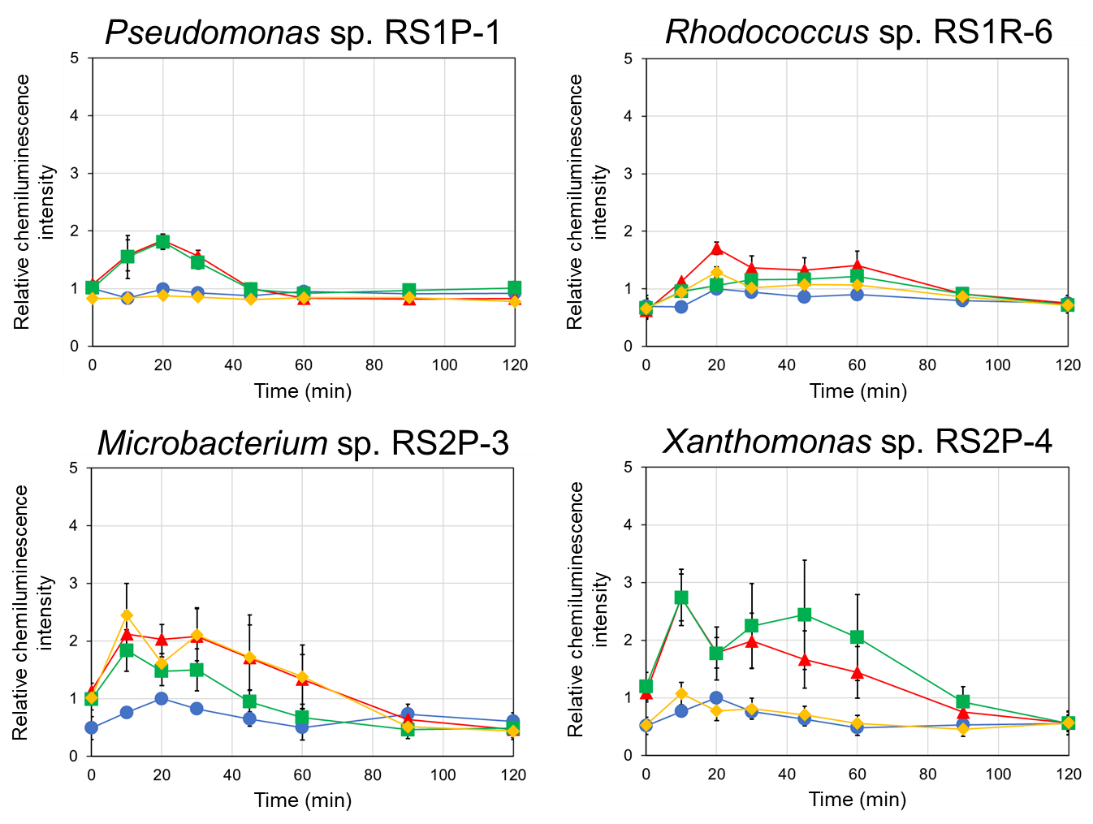


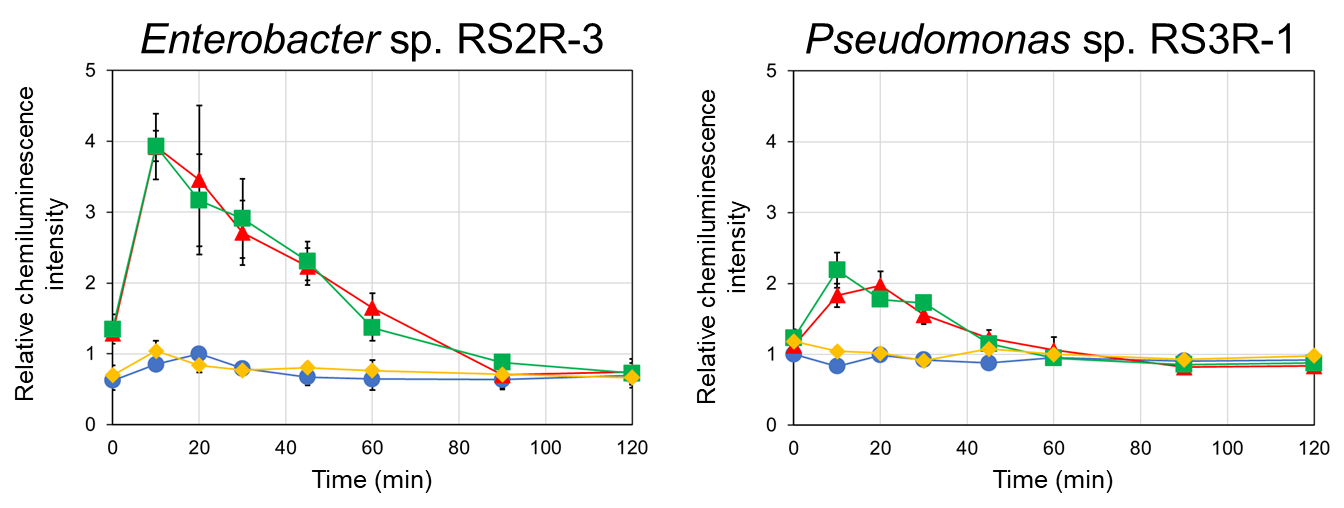


**Fig. S6** Localization of components that enhanced cryptogein-induced ROS production. BY-2 cells were co-incubated with bacterial cell culture solution (∆) or the cells (precipitate, □) or extracellular components (supernatant, ◊) after centrifugation of the solution, or subjected to mock treatment (only a mixture of medium and buffer, ○), and then cryptogein was added. ROS production was monitored based on chemiluminescence. The maximum value of the mock control was expressed as 1.0, and relative chemiluminescence intensity is shown. Average values ± SE from three independent experiments are presented.


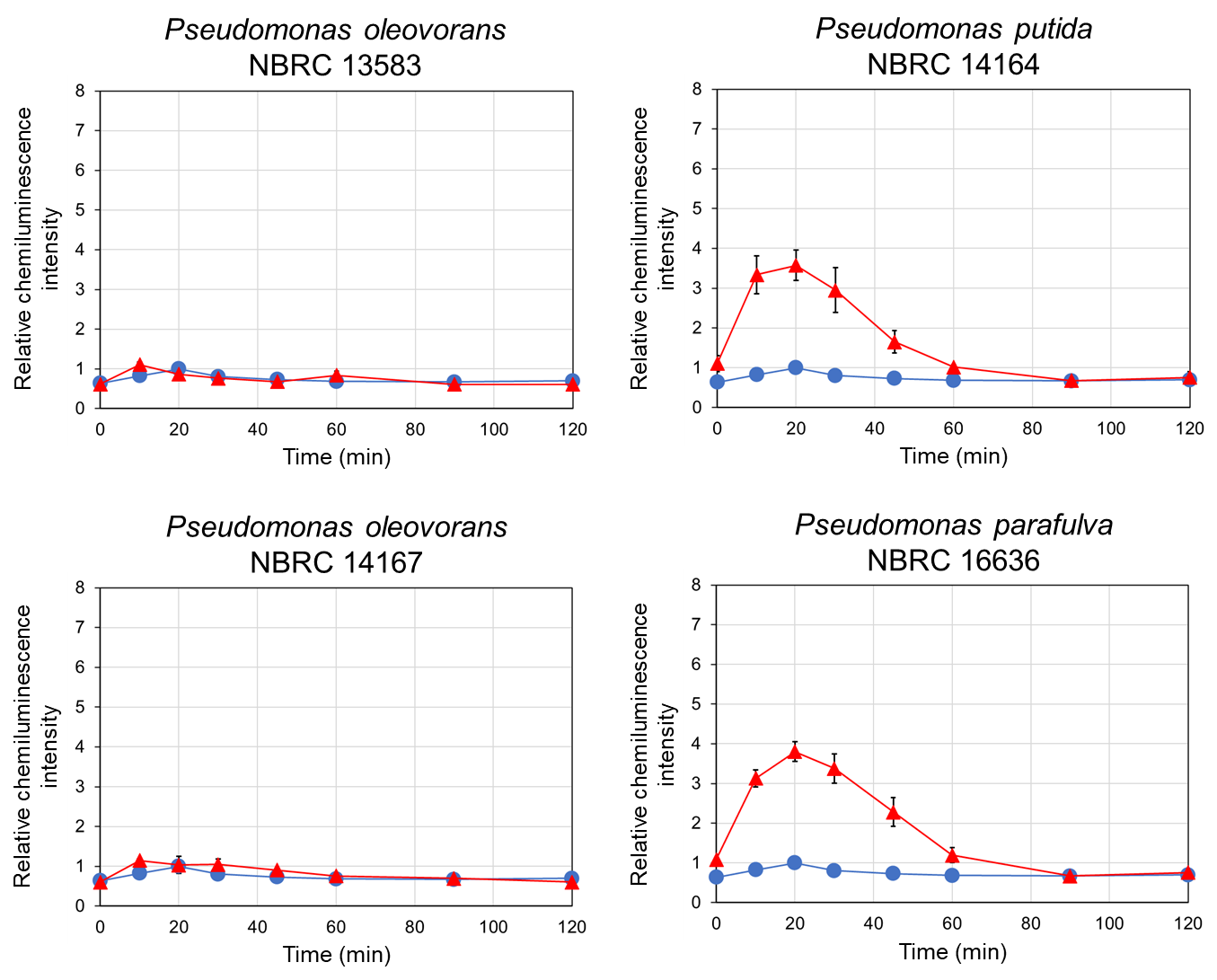


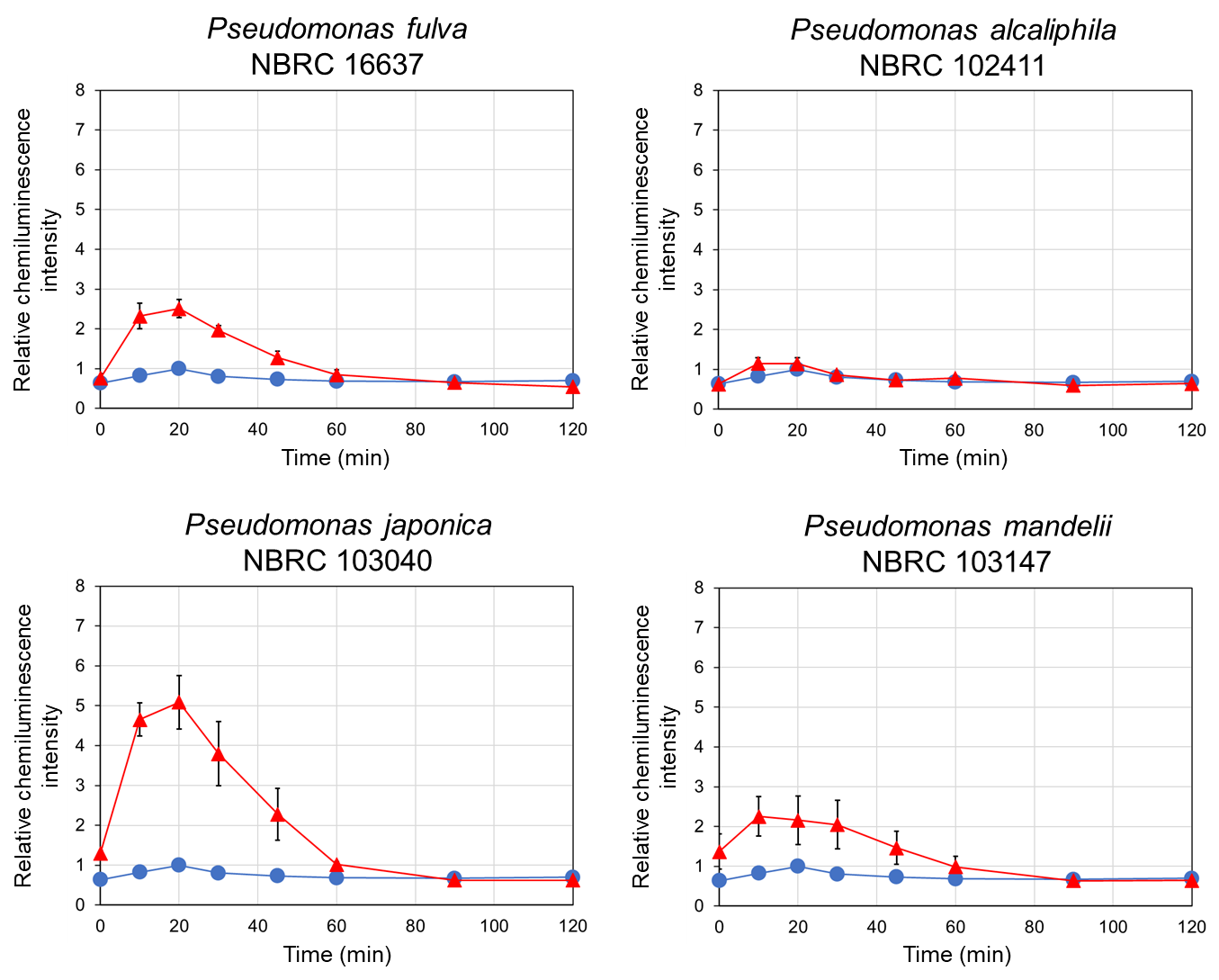


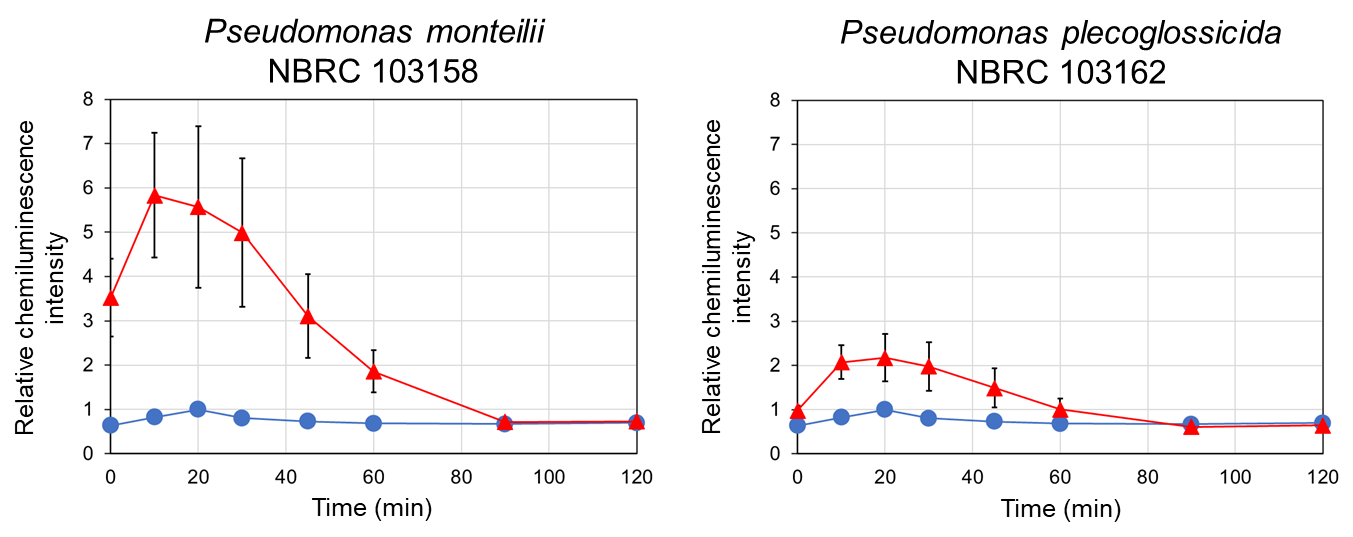


**Fig. S7.** Cryptogein-induced ROS production in BY-2 cells co-incubated with *Pseudomonas* strains obtained from NBRC. BY-2 cells were co-incubated with bacteria of each strain (∆) or subjected to mock treatment (only a mixture of medium and buffer, ○), and then cryptogein was added. ROS production was monitored based on chemiluminescence. The maximum value of the mock control was expressed as 1.0, and relative chemiluminescence intensity is shown. Average values ± SE from three independent experiments are presented.


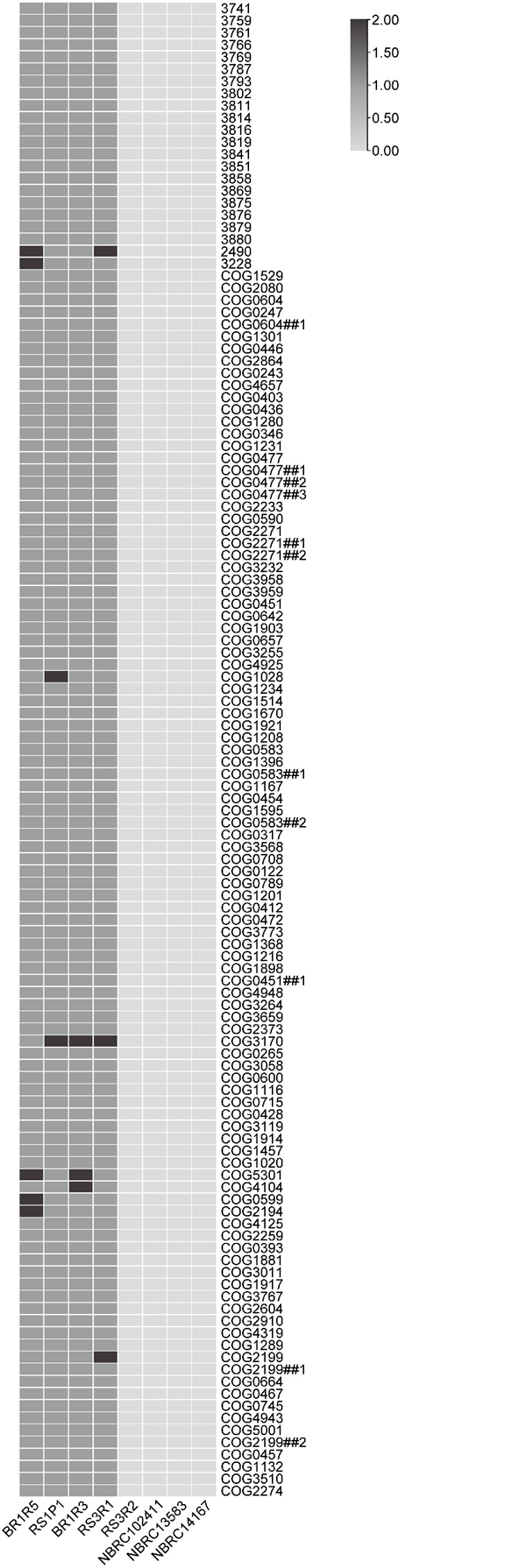


**Fig. S8.** Heatmap of Clusters of orthologous genes present in all ROS-enhancing strains that were absent in all non–ROS-enhancing strains.
